# Organ doses of the fetus from external environmental exposures

**DOI:** 10.1007/s00411-020-00891-6

**Published:** 2021-02-16

**Authors:** Nina Petoussi-Henss, Daiki Satoh, Helmut Schlattl, Maria Zankl, Vladimir Spielmann

**Affiliations:** 1 Institute of Radiation Medicine, Helmholtz Zentrum München, German Research Center for Environmental Health (GmbH), Ingolstaedter Landstr. 1, 85764 Neuherberg, Germany; 2 Nuclear Science and Engineering Center, Japan Atomic Energy Agency, Tokai-mura, Ibaraki 319-1195 Japan; 3 BfS, German Federal Office for Radiation Protection, Oberschleissheim, Germany

**Keywords:** External environmental exposure dosimetry, Fetus, Pregnant

## Abstract

This article presents nuclide-specific organ dose rate coefficients for environmental external exposures due to soil contamination assumed as a planar source at a depth of 0.5 g cm^−2^ in the soil and submersion to contaminated air, for a pregnant female and its fetus at the 24th week of gestation. Furthermore, air kerma free-in-air coefficient rates are listed. The coefficients relate the organ equivalent dose rates (Sv s^−1^) to the activity concentration of environmental sources, in Bq m^−2^ or Bq m^−3^, allowing to time-integrate over a particular exposure period. The environmental radiation fields were simulated with the Monte Carlo radiation transport codes PHITS and YURI. Monoenergetic organ dose rate coefficients were calculated employing the Monte Carlo code EGSnrc simulating the photon transport in the voxel phantom of a pregnant female and fetus. Photons of initial energies of 0.015–10 MeV were considered including bremsstrahlung. By folding the monoenergetic dose coefficients with the nuclide decay data, nuclide-specific organ doses were obtained. The results of this work can be employed for estimating the doses from external exposures to pregnant women and their fetus, until more precise data are available which include coefficients obtained for phantoms at different stages of pregnancy.

**Electronic supplementary material:**

The online version contains supplementary material available at 10.1007/s00411-020-00891-6

## Introduction

The monitoring of radiation doses received by a pregnant woman as well as its embryo and fetus due to naturally or anthropogenic radiation is of great interest to the public as well as to national and international organizations. This is important for members of the public and for workers who are occupationally exposed. The determination of the absorbed dose to the unborn child is a prerequisite for risk estimate calculations. The effects of exposure to radiation on the fetus depend on the time of exposure with respect to the time of conception and the amount of absorbed dose (Stovall et al. 1995; ICRP 2000, 2003).

The first days after a nuclear accident, both inhalation and external exposure from radionuclides in the air are important pathways. Ingestion could possibly play an important role after a certain period, if appropriate and quick restrictions for some foodstuffs are not taken. This was the case after the Chernobyl accident and the internal doses to the public in some regions were estimated to be comparable to the external doses. On the other hand, external exposure from radionuclide deposition on the ground is significant for a longer time than the internal exposure, as after the nuclear power plant accident in Fukushima Prefecture, Japan in March of 2011 (UNSCEAR 2013).

To evaluate the organ doses for external exposure to environmental radionuclides, dose rate coefficients are used, based on measured or predicted environmental activity concentration or measured air kerma free-in-air rate or ambient dose equivalent rate. Nuclide-specific dose rate coefficients depend on the exposure geometry, density and elemental composition of the soil and air, the spatial variability of the activity concentration, the energy and intensity of emitted radiations, and the transport of these radiations in the environment and within the exposed individual.

The International Commission on Radiological Protection (ICRP) reported dose coefficients for internal environmental exposures (dose per unit intake, Sv Bq^−1^) for the offspring of women exposed to radionuclides during or before pregnancy (ICRP 2001). These dose estimates include in utero doses to the embryo and fetus for acute or chronic intakes of radionuclides, by inhalation or ingestion, at different times before or during pregnancy. The accompanying papers by Stather et al. (2003) and Phipps et al. (2003) concluded that for most radionuclides, doses to the fetus will be lower than doses to the adult. However, for a few radionuclides, the dose to the fetus can exceed that to the mother.

Recently, age-dependent dose coefficients for external environmental exposures have been evaluated by ICRP for the ages represented by the ICRP reference phantoms: adult, 15-year-old (i.e., adolescent), and pediatric, i.e., newborn, 1-year-old, 5-year-old, 10-year-old (ICRP 2020). These are especially important for dose evaluation in the environment where wide range of the general public could be potentially exposed. Pregnant females and their fetuses have not been considered yet by the ICRP and the purpose of this work is, therefore, to provide dose coefficients for external environmental exposures of the pregnant female and fetus. Calculation of the coefficients requires the simulation of the environmental field (i.e., density and composition of ground, radionuclide distribution), the model of the body, i.e., the anthropomorphic phantom, and the simulation of the radiation transport in the human body.

To estimate the doses of a human, anthropomorphic phantoms are needed to represent the anatomy. A review of different types of anthropomorphic phantoms developed for radiation protection purposes can be found in Bolch et al. (2010) and Xu (2014).

In the following, some of the developed phantoms of mother and embryo or fetus will be mentioned, but the list might not be complete: Stabin et al. (1995) and Chen (2004) introduced the first mathematical stylized fetus phantoms for each gestational trimester. As the phantom construction technology evolved, the second generation of human phantoms appeared, the voxel phantoms, based on medical data of real persons, offering a better anatomical representation of organ location and shape. Shi and Xu (2004) developed a voxel phantom of a fetus at the 30th week of gestation. Becker et al. (2008) segmented a 24 week of gestation fetus from an abdominal magnetic resonance (MR) image of a patient and inserted this into the abdominal region of the ICRP reference female—see the section “Methods”.

The third generation of phantoms, called mesh or hybrid phantoms, is a combination of the above two techniques: the basis are medical data of real persons which are then modified with 3D surface modeling technologies to achieve an improved resolution and representation of organs and flexibility of shape. Xu et al. (2007) developed phantoms of pregnant women and their fetuses to represent the three gestational periods of 3, 6 and 9 months as specified by the ICRP for the reference individual (ICRP 2002). Maynard et al. developed a family of hybrid phantoms of the pregnant female (2011) and fetus (2014). These phantoms are ICRP Publication 89 (ICRP 2002) compliant reference phantoms and will be used by the ICRP for calculating reference dose coefficients. Moreover, Maynard et al. (2015a, b) developed Urals-based series of fetal and maternal models employed for fetal dosimetry to support the Techa river and Ozyorsk offspring cohorts’ epidemiological studies. Recent developments include hybrid pregnant and/or fetal phantoms for nuclear medicine applications: (Hoseinian-Azghadi et al. 2014; Xie and Zaidi 2016; Motavalli et al. 2016; Makkia et al. 2019).

At present, absorbed doses in the organs of the human body due to radiation fields are assessed using Monte Carlo radiation transport codes. The estimated organ doses are expressed as organ dose rate coefficients given as organ dose rates per unit of a measurable quantity. For environmental fields, this quantity is environmental activity concentration, or external dose rate measurement such as air kerma free-in-air or ambient dose equivalent. As this publication deals with environmental exposures of photons which have a radiation weighting factor, *w*_R_, equal to unity, the equivalent dose rate coefficients are numerically equal to their corresponding absorbed dose rate coefficients.

## Methods

### The environmental field

In the environment, exposures can be very variable and an exact simulation of all scenarios is not feasible. For the present work, two environmental exposure scenarios are considered, which are supposed to approximate the two most typical idealized exposure conditions:

1. Exposure from ground contamination (referred also as soil contamination) represented by a uniformly distributed monoenergetic planar source in the ground at a depth of 0.5 g cm^−2^: this depth is representative of the surface roughness and applicable in the first year following airborne deposition, by assuming an infinite plane source in the soil (ICRU 1994; ICRP 2020). Ground source (contamination of soil, concrete, asphalt etc.) is the most significant source of exposure in large-scale accidents since radionuclide deposition continues to expose the public for a long time and at large areas.
2. Exposure from a radioactive cloud, i.e., submersion to contaminated air—represented by a uniformly distributed monoenergetic semi-infinite volume source in the air: the assumed exposure geometry models the radioactive cloud, by assuming a homogeneous contamination of the air up to a height of 1000 m above a smooth air–ground interface. As radiation field characteristics in the air could change in various ways according to meteorological and other conditions as well as with time, the submersion model would be the most plausible model that can be applied to any source condition in the air.

For both sources mentioned above, photon emissions were considered. The soil was assumed as planar air–ground interface and scatter and absorption of the radiation fields in both air and ground were considered in the calculations.

**Fig. 1:**
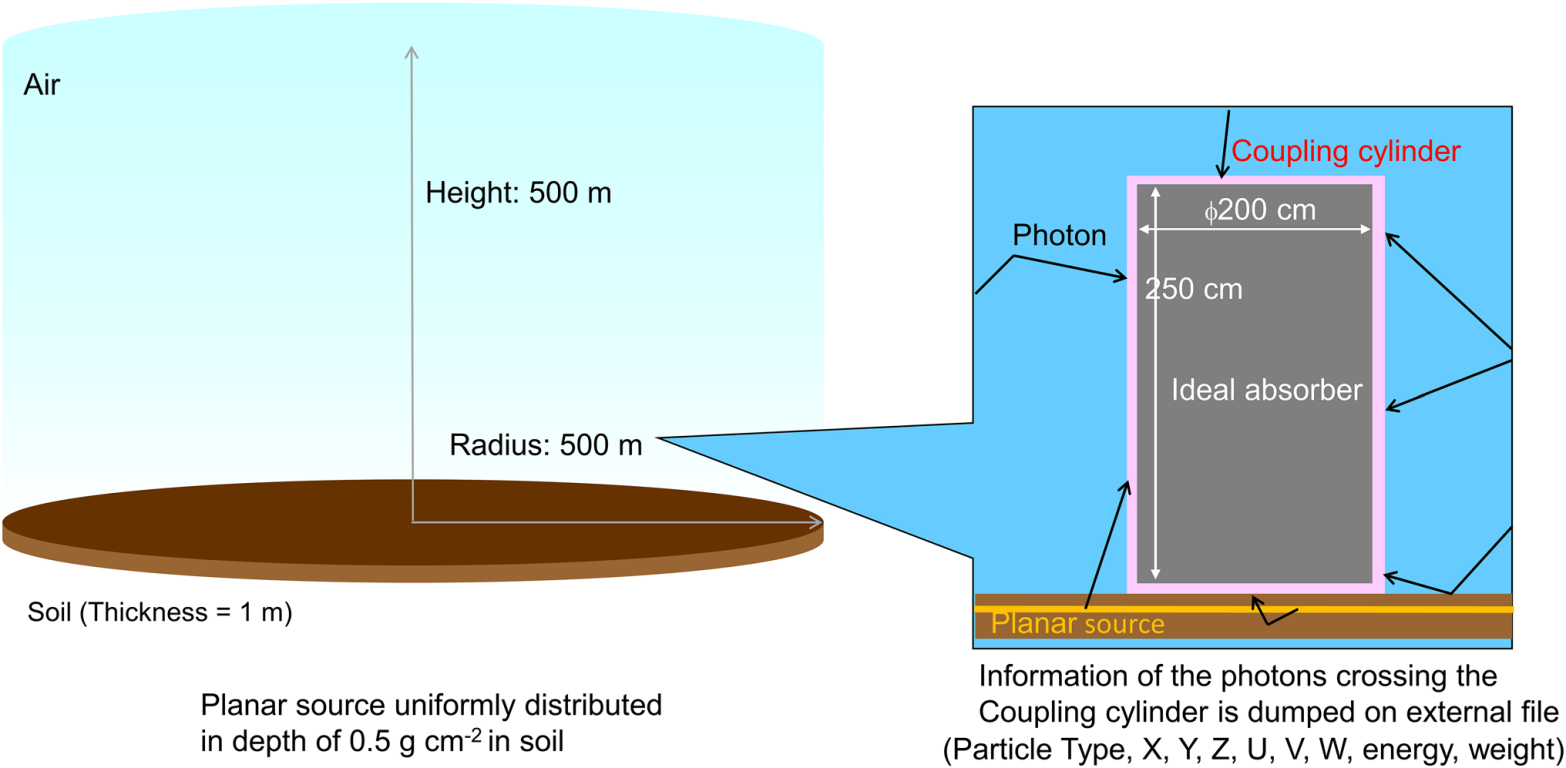
Depiction of the geometry simulating the environmental field for soil contamination

The radiation field modeling contamination of the soil was simulated using the Monte Carlo code PHITS (Particle and Heavy Ion Transport code System) (Sato et al. 2013). Photons were transported, and secondary electrons released by photon interactions were not followed. This is because the secondary electrons lose their energies continuously and stop within a short distance in the environmental media. However, bremsstrahlung photons generated by secondary electrons have energies that distribute from nearly zero to the maximum energy of the secondary electrons and could travel along long ranges. The production of the bremsstrahlung photons, and their energy and emission angle were sampled at the interaction point based on a thick-target bremsstrahlung approximation model (MCNP 2003). Figure 1 shows schematically the geometry of the simulation, which is composed of a right circular cylinder consisting of a layer of air and soil with a thickness of 500 and 1 m, respectively. The 500 m radius of the cylinder corresponds to about five times the mean free path of 0.6 MeV photons in air.

The radiation field simulating submersion to contaminated air has been previously determined by Saito et al. (Saito et al. 1990; Saito and Jacob 1995; Petoussi-Henss et al. 2012) using the Monte Carlo code YURI (Saito and Moriuchi 1985). The source is the semi-infinite cloud containing a uniformly distributed activity concentration of the radioactive source and the individual is standing on an uncontaminated flat surface of infinite area, in a large volume of uniformly contaminated air.

For both source types, the radiation fields were computed for incident monoenergetic photon energies in the range of 0.015–10 MeV, so as to cover the wide energy spectra of naturally occurring and man-made radionuclides. The accuracy of PHITS and YURI codes has been well validated for photon and electron transport calculations (Saito and Moriuchi 1985; Saito et al. 1990; Sato et al. 2013).

Table 1 shows the density and elemental composition of air and soil assumed in the present calculations. A standard composition of soil is difficult to be defined, as this varies from place to place in elemental composition both qualitatively and quantitatively. For this work, the values were obtained from the data for soil (Type 1) provided by the International Commission on Radiation Units and Measurements (ICRU) (ICRU 1994) and dry air from the National Institute of Standards and Technology (NIST) (Berger et al. 2005), respectively. The densities of soil and air were considered to be 1 g cm^−3^ and 1.2 × 10^–3^ g cm^−3^, respectively. In real situations, the soil densities are between 0.8 and 1.6 g cm^−3^ and mostly higher than 1 g cm^−3^ and could differ depending on site and soil depth; however, this variation does not significantly affect the relation of source intensity to the radiation field in air, if source depth is expressed in terms of g cm^−2^. Moreover, it has been previously demonstrated that variations in soil density do not significantly affect the simulated photon fields incident on the phantoms (Saito and Jacob 1995).

**Table 1:** Density and elemental composition of air (Berger et al. 2005) and soil (ICRU 1994)

Material	Density (g cm^−3^)	Elemental composition (weight %)							
		H	C	N	O	Al	Si	Ar	Fe
Air	1.2 × 10^–3^	–	0.01	75.53	23.18	–	–	1.28	–
Soil	1.0	2.20	–	–	57.50	8.50	26.20	–	5.60

**Fig. 2:**
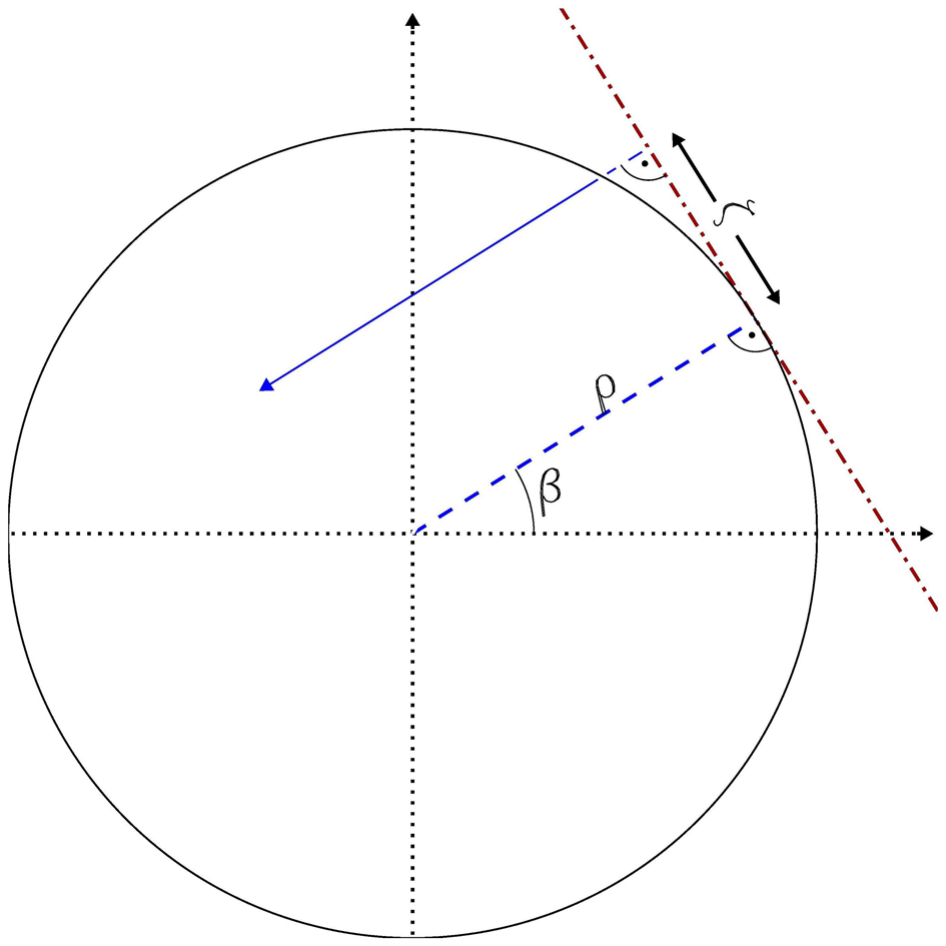
Coordinates describing the position and direction of a particle (blue arrow) emitted at the side of the coupling cylinder in the horizontal plane

From the Monte Carlo calculations in the environmental field, individual particles were recorded at the surface of a virtual cylinder called the “coupling cylinder”. This cylinder is positioned on the ground concentric with the simulation geometry, as shown in Fig. 1 (right) and different phantoms may be placed inside, if calculations for different subjects are required. For soil contamination, the diameter of the cylinder is 2 m, and its height is 2.5 m, whereas for submersion to contaminated air the diameter of the cylinder is 0.6 m, and its height is 2 m. The spatial coordinates, momentum, energy, angular information and Monte Carlo weight of the photons hitting this cylinder are recorded. The space inside the coupling cylinder is considered to be an ideal absorber, i.e., the Monte Carlo code terminates the transport of the particle when it enters this region. Approximately, 300–4.7 million photons were recorded on the cylinder surface, the number of particle histories followed being lower for higher energies to reduce the computational time. The data were recorded to an external file in ASCII format, containing energy/angular information (phase space) for radiation particles striking this cylinder to be used for the organ dose calculations within the phantom. Analogue to Saito et al. (1991), the phase space information was used to create probability density functions *p*_S_*(*cos*ϑ, h, E)* and *p*_L_*(*cos*ϑ, E)* describing the particle emission distribution on the sides and lids of the coupling cylinder, respectively, where *ϑ* is the polar angle of the particle direction, *h* the height of the emission position on the cylinder side, and *E* the particle energy. For this purpose, particles having cos*ϑ, h*, and *E* within predefined bins are counted and normalized, yielding discrete probability density functions. Bin sizes of 0.1 and 10 cm in cos*ϑ* and *h*, respectively, were chosen and the energy follows in all decades the grid of 1.0, 1.5, 2.0, 3.0, 4.0, 5.0, 6.0, 7.0, 8.0, and 9.0. It is worth noticing that, since the environmental field should be invariant to rotation or translation, the same must be valid for the emission field of the coupling cylinder. Therefore, it can be assumed that the particle emission from the cylinder sides is uniform in the horizontal plane when described by the quantities *ζ* and *β* (see Fig. 2, *ρ* is the radius of the cylinder). On the cylinder lids, the positions and azimuthal directions of the emission are uniformly distributed in (*ζ*, *ρ)* and in *β*, respectively*.* It has been verified that the phase space information indeed obeys these conditions. Thus, for the creation of the probability density function, the phase space information about *β*, *ζ* and *ρ* (on the cylinder lids) can be ignored, leading to statistical uncertainties of *p*_S_*(*cos*ϑ, h, E)* and *p*_L_*(*cos*ϑ, E)* which are considerably lower than the respective ones of *p*_S_*(β*, *ζ*, cos*ϑ, h, E)* and *p*_L_*(β,ζ*, *ρ*, cos*ϑ, E)* would have been.

### The computational anthropomorphic phantom “Katja” and its fetus

A whole-body voxel phantom of a pregnant woman at the 24th week of gestation was previously constructed at the Helmholtz Zentrum München. The model called “Katja” (Becker et al. 2008; Zankl 2010), integrates two voxel phantoms, one of a fetus and the ICRP adult female reference phantom (ICRP 2009a). The fetus was segmented from a magnetic resonance imaging dataset of the abdominal and pelvic regions of a female patient in the 24th week of pregnancy. The organs of the fetus that could be well visualized and segmented are brain, brain liquid, skull, eyes, eye lenses, skin, soft tissue, spinal cord, lungs, liver, kidneys, heart, gall bladder and stomach. Since the phantom of the fetus stemmed from a magnetic resonance scan, it was not possible to identify any more bones than spine and skull. The pelvis and abdomen of the ICRP reference female voxel phantom (ICRP 2009a) were then anatomically adjusted to incorporate the fetus and placenta. These were then virtually “transplanted” to the body of the ICRP reference female voxel model, creating a whole-body model of a pregnant woman suitable for calculations in radiation protection (Table 2).

**Table 2:** Characteristics of the voxel phantom “Katja”

Name	Age	Mass (kg)	Height (cm)	Voxel dimensions (mm^3^)	Number of organs
“Katja” + embryo	43 years 24 week of gestation	62.3	163	1.77 × 1.77 × 4.84	136 + 19

### Monte Carlo calculations of the organ equivalent dose rate coefficients for monoenergetic photons

For the above-described exposure geometries of environmental media, i.e., contaminated soil and air, Monte Carlo calculations of organ absorbed dose rate coefficients were performed with a user code which employs EGSnrc, version v4-2-3-0 (Kawrakow et al. 2009). For more details on the code used, see Schlattl et al. (2007). In the computations of this work, the tracks of primary and secondary photons are followed down to kinetic energies of 2 keV, those of secondary electrons down to 20 keV. Electrons are assumed to deposit their energy continuously.

**Fig. 3:**
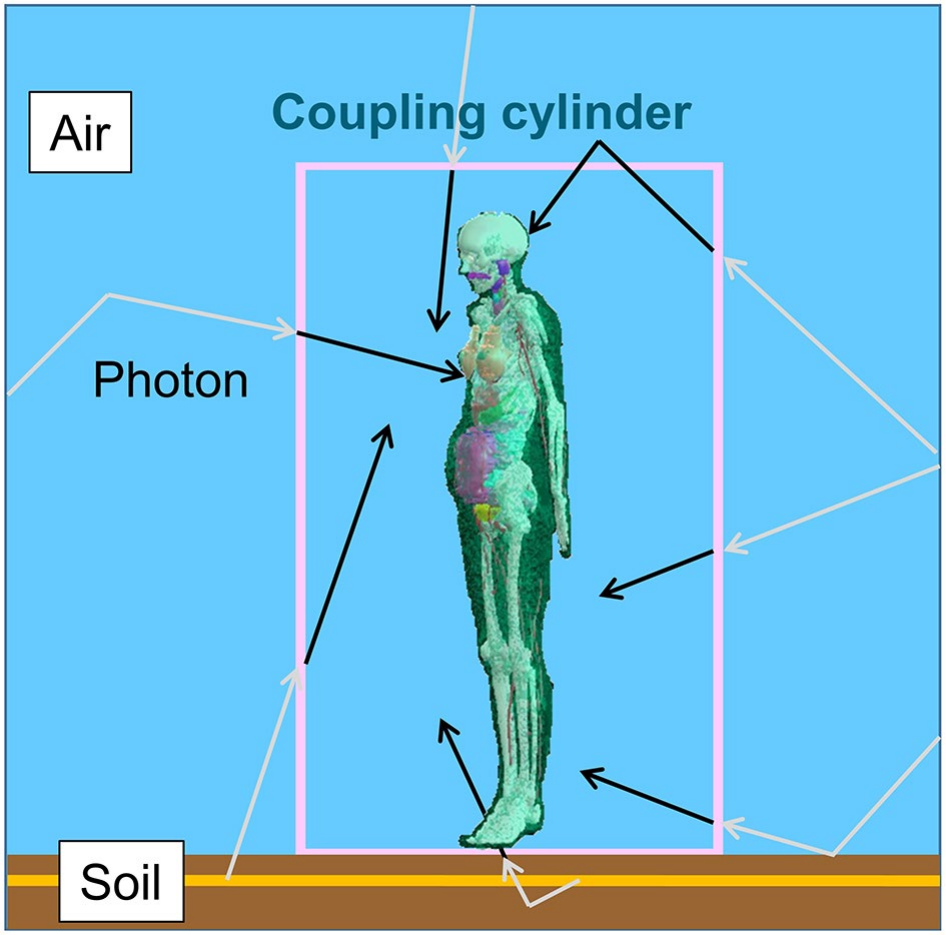
Depiction of the geometry simulating the organ dose calculations

The phantom, placed inside the air-filled coupling cylinder, stands on the soil, simulated as a planar air/ground interface. The application of the coupling cylinder has been previously used by many researchers as it significantly improves the calculation efficiency and statistical accuracy of the computed results, because the same radiation fields recorded at the surface of the cylinder can be repeatedly used for different exposed computational phantoms if this is required (Saito et al. 1991; Petoussi-Henss et al. 2012; Satoh et al. 2015; Bellamy et al. 2019; Kofler et al. 2019; ICRP 2020). The particle histories are restarted from the surface of the coupling cylinder irradiating the phantom. Figure 3 shows the schematic representation of the geometry simulating the organ dose calculations. The density probability functions based on monoenergetic incident environmental photon sources are employed, binned in a manner described in “The environmental field”. Calculations for photon energies between 0.015 and 10 MeV have been considered and for each irradiation, 7.5–200 million photon histories were simulated. The coefficients of variance were generally around 0.2% for large organs and lower than 3% for most of the small organs.

For comparison purposes, organ dose calculations were also performed for the above-mentioned geometries and the ICRP female reference phantom (ICRP 2009a). Moreover, air kerma free-in-air coefficient rates were obtained at height of 1 m above the ground. In the following text, the quantity air kerma free-in-air will be referred to simply as ‘air kerma’. The air kerma is a basic quantity related to radiation intensity, and in natural environments is substantially equivalent to the air absorbed dose which has been used in environmental monitoring for a long time to express dose rates in air (UNSCEAR 2008, 2000). Its value depends on the deposition density of radionuclides, initial attenuation of radiation in soil, radioactive decay, soil properties, water (i.e., moisture) content in soil, vertical migration of radionuclides into soil, and the presence of snow. The calculation was performed by modeling a 30 cm diameter sphere filled with and surrounded by air, placed at 1 m above the ground and by sampling the particles entering the sphere. The photon fluence rate scored at the air sphere was then converted to an air kerma rate using the dose coefficients given in ICRP Publication 74 (ICRP 1996).

### Nuclide-specific dose rate coefficients

To define the dose rate coefficients from radionuclides in the environment, dose rate coefficients for monoenergetic photons were employed by scaling them to the emissions of the radionuclides of interest, using the nuclear decay data contained in ICRP Publication 107 (ICRP 2008). Only photons, including bremsstrahlung, and electrons emitted by the radionuclides are sufficiently penetrating to traverse the overlying tissues of the body and contribute to the dose to tissues and organs of the body.

Radionuclide-specific equivalent dose rate coefficients, $\dot{h}_{T}^{S}$ for tissue *T* and exposure mode, *S*, can be expressed as:

1 $$ \dot{h}_{T}^{S} = \sum\nolimits_{R} {w_{R} } \left[ {\sum\nolimits_{i} {Y_{R,i} \left( {E_{i} } \right)\dot{d}_{T,R}^{S} \left( {E_{i} } \right)} + \int_{0}^{\infty } {Y_{R} \left( E \right)\dot{d}_{T,R}^{S} \left( E \right)dE} } \right], $$

where *R* indicates the radiation type, and ${w}_{R}$ the radiation weighting factor of the radiation type *R*. The outer summation is over radiation types (i.e., photons and electrons) emitted from a radionuclide. ${Y}_{R,i}$ is the yield of *i*-th radiation of type *R* per decay (or nuclear transformation) having discrete energy ${E}_{i}$ emitted by a nuclear decay of the radionuclide in summation over discrete energy emissions and ${\dot{d}}_{T,R}^{S}\left({E}_{i}\right)$ is the organ absorbed dose rate coefficient at the energy ${E}_{i}$ for tissue, *T*, radiation type, *R*, and exposure mode, *S*, for monoenergetic values. The first term within the major bracket sums over all radiations emitted with discrete energies by the nuclear decay. In the integration of the second term, ${Y}_{R}\left(E\right)$ is the yield expressing the source particle spectrum per decay with dimension (MeV^−1^) and ${\dot{d}}_{T,R}^{S}\left(E\right)$ the absorbed dose rate coefficient, at the energy *E* within a continuous energy spectrum of beta emissions.

For the interpolation of dose coefficients, a piecewise cubic Hermite function (Fritsch and Carlson 1980) was used in a log-linear space. As the coefficients for monoenergetic radiations obtained by Monte Carlo calculations addressed photons of 0.015 MeV and higher energy, the values at energies less than 0.015 MeV were set to zero.

As already stated, for contamination in the soil, very few electrons could traverse the soil and reach the human body. For this reason, no electron fields are considered, however, bremsstrahlung photons produced by electrons slowing down in the soil or air could be sufficiently penetrating to contribute mainly to the dose to the skin (i.e., of the mother). Bremsstrahlung energy is distributed from zero up to the initial electron energy. Hence, for a large collection of monoenergetic electrons, a continuous bremsstrahlung energy spectrum is generated. Although the bremsstrahlung yield is small (only about 0.2% for a 1.0 MeV electron in air) for pure beta emitters, it can be the only source of radiation sufficiently penetrating to reach radiosensitive tissues lying deeply in the body (Bellamy et al. 2019). Therefore, the bremsstrahlung photons were taken into account using respective bremsstrahlung spectra taken from Eckerman and Ryman (1993). Similarly, the bremsstrahlung contribution to the dose coefficients for air submersion was considered.

Radionuclide-specific organ equivalent dose rate coefficients were evaluated for 1252 radionuclides of 97 elements compiled in *Publication 107* (ICRP 2008) for the geometries described above, i.e., soil contamination at planar source at a depth of 0.5 g cm^−2^ and air submersion. It should be noted that the dose rate coefficients provided are calculated for the indicated radionuclide only and do not include radiations from daughter nuclides. If the decay of selected radionuclide yields radioactive decay products (progeny), the dose contribution of a radionuclide and its progeny need to be evaluated considering the production and decay of radioactive progeny and differences in environmental behavior of the parent and daughter nuclides. In ICRP Publication 107 (2008), a summary information on the nuclear transformation of radionuclides can be found. Such information is necessary for evaluation of the dose rate at a specified time and the dose integrated over a specified period (Eckerman and Ryman 1993; Bellamy et al. 2019; ICRP 2020).

## Results and discussion

Table 3 shows the calculated values of air kerma at 1 m height above the ground for soil contamination at a depth of 0.5 g cm^−2^, calculated for this work and for submersion in contaminated air, previously calculated by Saito et al. (Saito et al. 1990) for monoenergetic photons. Note that Saito et al. used fluence-to-air-kerma conversion coefficients of Hubbell (1982), which are up to 4% higher for photon energies below 0.060 and above 8 MeV, than the values of Seltzer (1993) used in this work.

**Table 3:** Air kerma coefficient rates for radionuclides distributed at a depth of 0.5 g cm^−2^ in the soil and for submersion to contaminated air, estimated at 1 m above ground

E (MeV)	Soil contamination	Air submersion*
	$\dot{k}_{a}$ (Gy s^−1^ Bq^−1^ m^2^)	$\dot{k}_{a}$ (Gy s^−1^ Bq^−1^ m^3^)
0.015	7.29E−19	1.47E−15
0.020	7.42E−18	1.71E−15
0.030	2.60E−17	2.21E−15
0.040	3.51E−17	2.40E−15
0.050	3.94E−17	2.81E−15
0.060	4.53E−17	3.31E−15
0.070	5.09E−17	3.79E−15
0.080	5.78E−17	4.36E−15
0.100	7.41E−17	5.55E−15
0.150	1.20E−16	8.68E−15
0.200	1.68E−16	1.20E−14
0.300	2.62E−16	1.87E−14
0.400	3.51E−16	
0.500	4.39E−16	3.21E−14
0.600	5.22E−16	
0.700		4.56E−14
0.800	6.78E−16	
1.000	8.26E−16	6.58E−14
1.500	1.15E−15	9.80E−14
2.000	1.45E−15	1.32E−13
3.000	1.98E−15	1.96E−13
4.000	2.47E−15	
5.000	2.93E−15	
6.000	3.37E−15	3.19E−12
8.000	4.19E−15	
10.000	5.07E−15	4.81E−12

^*^From Saito et al. (1990)

Tables 4 and 5 show the equivalent dose rates for organs of the fetus for soil contamination and air submersion, respectively, for monoenergetic photon fields.

**Table 4:** Organ equivalent dose rate coefficients (Sv s^−1^ Bq^−1^ m^2^) of fetus (24th week of gestation) for monoenergetic photon sources distributed at a depth of 0.5 g cm^−2^ in the soil

Organ/tissue	Energy (MeV)									
	0.015	0.02	0.03	0.04	0.05	0.06	0.07	0.08	0.1	0.15
Skin	7.00E−23	2.98E−20	1.87E−18	8.19E−18	1.58E−17	2.37E−17	3.09E−17	3.79E−17	5.11E−17	8.16E−17
Eyes	0.00E + 00	2.76E−21	1.04E−18	5.69E−18	1.26E−17	1.96E−17	2.66E−17	3.34E−17	4.63E−17	7.96E−17
Lungs	4.37E−24	1.17E−20	1.62E−18	7.98E−18	1.58E−17	2.38E−17	3.10E−17	3.78E−17	5.11E−17	8.22E−17
Heart	0.00E + 00	3.89E−21	1.18E−18	6.82E−18	1.50E−17	2.27E−17	2.92E−17	3.62E−17	4.94E−17	8.10E−17
Kidneys	7.84E−24	1.58E−20	1.72E−18	8.32E−18	1.66E−17	2.47E−17	3.24E−17	3.92E−17	5.33E−17	8.34E−17
Liver	4.48E−24	1.33E−20	1.67E−18	8.22E−18	1.61E−17	2.41E−17	3.17E−17	3.85E−17	5.20E−17	8.33E−17
Stomach	0.00E + 00	1.61E−21	8.11E−19	5.56E−18	1.24E−17	2.06E−17	2.78E−17	3.22E−17	4.66E−17	7.58E−17
Brain	2.77E−23	2.35E−20	1.73E−18	7.56E−18	1.51E−17	2.31E−17	3.01E−17	3.74E−17	5.01E−17	8.15E−17
Skeleton	5.12E−23	3.67E−20	2.92E−18	1.31E−17	2.56E−17	3.71E−17	4.69E−17	5.61E−17	7.08E−17	1.03E−16
Total body	2.61E−23	1.79E−20	1.63E−18	7.77E−18	1.56E−17	2.36E−17	3.09E−17	3.79E−17	5.12E−17	8.22E−17

Organ/tissue	Energy (MeV)									
	0.2	0.3	0.4	0.5	0.6	0.7	0.8	1	1.1	1.3
Skin	1.12E−16	1.72E−16	2.32E−16	2.92E−16	3.53E−16	4.11E−16	4.70E−16	5.87E−16	6.36E−16	7.53E−16
Eyes	1.04E−16	1.62E−16	2.29E−16	2.86E−16	3.30E−16	3.89E−16	4.48E−16	5.74E−16	6.05E−16	7.86E−16
Lungs	1.13E−16	1.72E−16	2.33E−16	2.93E−16	3.51E−16	4.08E−16	4.65E−16	5.90E−16	6.29E−16	7.52E−16
Heart	1.08E−16	1.66E−16	2.30E−16	2.84E−16	3.47E−16	4.08E−16	4.69E−16	5.73E−16	6.07E−16	7.60E−16
Kidneys	1.14E−16	1.73E−16	2.38E−16	2.98E−16	3.59E−16	4.17E−16	4.75E−16	5.85E−16	6.30E−16	7.50E−16
Liver	1.13E−16	1.72E−16	2.32E−16	2.95E−16	3.54E−16	4.14E−16	4.73E−16	5.79E−16	6.31E−16	7.59E−16
Stomach	1.09E−16	1.69E−16	2.17E−16	2.80E−16	3.42E−16	4.02E−16	4.62E−16	5.60E−16	6.21E−16	7.04E−16
Brain	1.12E−16	1.70E−16	2.27E−16	2.89E−16	3.45E−16	4.01E−16	4.58E−16	5.78E−16	6.25E−16	7.40E−16
Skeleton	1.31E−16	1.90E−16	2.44E−16	3.03E−16	3.62E−16	4.17E−16	4.72E−16	5.96E−16	6.35E−16	7.57E−16
Total body	1.12E−16	1.71E−16	2.30E−16	2.91E−16	3.49E−16	4.07E−16	4.64E−16	5.83E−16	6.30E−16	7.48E−16

Organ/tissue	Energy (MeV)									
	1.5	2	3	4	5	6	7	8	10	
Skin	8.54E−16	1.11E−15	1.60E−15	2.06E−15	2.48E−15	2.90E−15	3.23E−15	3.56E−15	4.34E−15	
Eyes	8.23E−16	1.04E−15	1.59E−15	2.04E−15	2.44E−15	2.88E−15	3.33E−15	3.78E−15	4.27E−15	
Lungs	8.48E−16	1.09E−15	1.61E−15	2.09E−15	2.50E−15	2.89E−15	3.27E−15	3.64E−15	4.30E−15	
Heart	8.26E−16	1.09E−15	1.60E−15	2.07E−15	2.48E−15	2.82E−15	3.18E−15	3.54E−15	4.17E−15	
Kidneys	8.55E−16	1.12E−15	1.61E−15	2.10E−15	2.43E−15	2.83E−15	3.21E−15	3.59E−15	4.37E−15	
Liver	8.46E−16	1.13E−15	1.59E−15	2.10E−15	2.48E−15	2.86E−15	3.20E−15	3.54E−15	4.32E−15	
Stomach	8.49E−16	1.09E−15	1.51E−15	2.10E−15	2.44E−15	2.96E−15	3.16E−15	3.36E−15	4.40E−15	
Brain	8.40E−16	1.10E−15	1.61E−15	2.05E−15	2.45E−15	2.95E−15	3.29E−15	3.62E−15	4.29E−15	
Skeleton	8.42E−16	1.09E−15	1.58E−15	2.05E−15	2.44E−15	2.90E−15	3.25E−15	3.60E−15	4.23E−15	
Total body	8.47E−16	1.11E−15	1.60E−15	2.07E−15	2.47E−15	2.90E−15	3.23E−15	3.57E−15	4.32E−15

**Table 5:** Organ equivalent dose rate coefficients (Sv s^−1^ Bq^−1^ m^3^) of fetus (24th week of gestation) for monoenergetic photon sources and air submersion

	Energy (MeV)									
Organ/Tissue	0.015	0.02	0.03	0.04	0.05	0.06	0.07	0.08	0.1	0.15
Skin	8.85E−19	1.67E−17	2.09E−16	5.56E−16	9.96E−16	1.48E−15	1.93E−15	2.40E−15	3.30E−15	5.41E−15
Eyes	0.00E + 00	1.95E−18	8.66E−17	3.22E−16	6.78E−16	1.10E−15	1.52E−15	1.98E−15	2.86E−15	4.75E−15
Lungs	6.97E−20	7.59E−18	1.89E−16	5.49E−16	1.01E−15	1.49E−15	1.92E−15	2.44E−15	3.35E−15	5.45E−15
Heart	0.00E + 00	2.88E−18	1.53E−16	4.78E−16	9.49E−16	1.43E−15	1.91E−15	2.35E−15	3.24E−15	5.32E−15
Kidneys	2.63E−19	1.55E−17	2.48E−16	6.62E−16	1.17E−15	1.71E−15	2.11E−15	2.64E−15	3.65E−15	5.74E−15
Liver	1.75E−19	1.16E−17	2.38E−16	6.37E−16	1.12E−15	1.65E−15	2.10E−15	2.62E−15	3.58E−15	5.73E−15
Stomach	0.00E + 00	2.10E−18	1.11E−16	4.22E−16	8.13E−16	1.30E−15	1.70E−15	2.18E−15	2.93E−15	5.09E−15
Brain	7.66E−21	1.52E−18	8.44E−17	3.26E−16	7.03E−16	1.12E−15	1.51E−15	1.97E−15	2.78E−15	4.70E−15
Skeleton	4.77E−20	5.36E−18	2.02E−16	6.79E−16	1.33E−15	2.00E−15	2.64E−15	3.25E−15	4.33E−15	6.66E−15
Total body	3.12E−19	9.39E−18	1.74E−16	5.08E−16	9.49E−16	1.43E−15	1.87E−15	2.35E−15	3.25E−15	5.34E−15

	Energy (MeV)									
Organ/tissue	0.2	0.3	0.5	0.7	1	1.5	2	3	6	10
Skin	7.50E−15	1.17E−14	2.03E−14	2.90E−14	4.36E−14	6.73E−14	9.40E−14	1.47E−13	3.08E−13	5.14E−13
Eyes	6.58E−15	1.00E−14	1.87E−14	2.69E−14	4.08E−14	5.91E−14	8.79E−14	1.51E−13	3.06E−13	4.84E−13
Lungs	7.47E−15	1.19E−14	2.02E−14	2.97E−14	4.37E−14	6.62E−14	9.59E−14	1.48E−13	3.02E−13	5.18E−13
Heart	7.28E−15	1.17E−14	1.98E−14	2.91E−14	4.36E−14	6.61E−14	9.23E−14	1.56E−13	3.21E−13	5.09E−13
Kidneys	7.96E−15	1.25E−14	2.13E−14	3.10E−14	4.45E−14	6.97E−14	9.95E−14	1.54E−13	3.16E−13	5.12E−13
Liver	7.83E−15	1.23E−14	2.12E−14	3.06E−14	4.51E−14	6.87E−14	9.81E−14	1.52E−13	3.12E−13	5.23E−13
Stomach	7.03E−15	1.17E−14	2.02E−14	2.83E−14	4.56E−14	6.57E−14	8.96E−14	1.44E−13	2.93E−13	4.93E−13
Brain	6.58E−15	1.05E−14	1.84E−14	2.66E−14	3.99E−14	6.15E−14	8.76E−14	1.41E−13	2.99E−13	5.01E−13
Skeleton	8.75E−15	1.29E−14	2.12E−14	2.95E−14	4.28E−14	6.55E−14	9.08E−14	1.44E−13	3.01E−13	4.98E−13
Total body	7.38E−15	1.16E−14	2.00E−14	2.88E−14	4.29E−14	6.66E−14	9.31E−14	1.46E−13	3.07E−13	5.12E−13

**Fig. 4:**
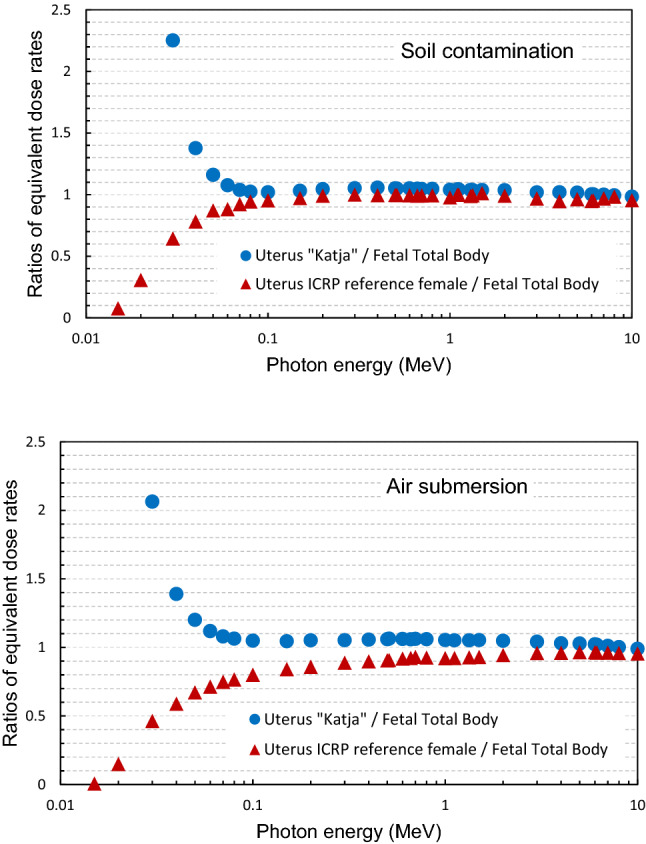
Ratios of the uterus equivalent dose rates of the pregnant phantom “Katja” to the equivalent fetal total body dose rate coefficients. The respective ratios of the uterus dose coefficient rates of the ICRP reference female phantom to the coefficients of fetus total body dose rates are also shown; exposures: soil contamination (top) and submersion in a radioactive cloud (bottom). For better visibility, some values for energies below 0.03 MeV have been omitted

Chen (2012) has shown that the dose to an embryo for the first weeks of pregnancy, even for the first trimester, can be approximated by the dose to the uterus. The results of the present study support this finding: Fig. 4 shows, for soil contamination (upper figure) and air submersion (lower figure), the ratios of the uterus equivalent dose rates of the pregnant phantom “Katja” to the equivalent fetal total body dose rate coefficients. It can be seen that the uterus dose of the pregnant phantom is conservatively approximating the dose to the fetus. For comparison purposes, the respective ratios of the uterus dose coefficient rates of the ICRP reference female phantom to the fetus total body dose rates are also shown. The higher doses of the uterus of the pregnant phantom, compared to the uterus of the non-pregnant, are due to the enlarged uterus of the pregnant phantom carrying the fetus, reaching more shallow positions and thus being less shielded by adipose tissue.

Table 6 shows, for some selected radionuclides and for both environmental geometries, the nuclide-specific dose rate coefficients for air kerma free-in-air at 1 m above the ground, the uterus dose of the pregnant model “Katja” as well as the total body dose of the fetus. For comparison purposes, the detriment-weighted dose of “Katja” is also shown. This has been estimated by considering the organs assigned a tissue weighting factor for the calculaltion of the effective dose as per ICRP Publication 103 (ICRP 2007) and computing the respective weighted sum.

**Table 6:** Nuclide-specific kerma rate, detriment-weighted dose rate and uterus equivalent dose rate coefficients of the pregnant model “Katja”, as well as fetal total body equivalent dose rate coefficients for a ground plane source at a depth of 0.5 g cm^−2^ and for submersion to contaminated air

	Soil contamination				Air submersion			
Nuclide	$\dot{k}_{a}$	Detriment weighted dose “Katja”	Uterus (“Katja”)	Total body (fetus)	$\dot{k}_{a}$a	Detriment weighted dose “Katja”	Uterus (“Katja”)	Total body (fetus)
	Gy s^−1^ Bq^−1^ m^2^	Sv s^−1^ Bq^−1^ m^2^	Sv s^−1^ Bq^−1^ m^2^	Sv s^−1^ Bq^−1^ m^2^	Gy s^−1^ Bq^−1^ m^3^	Sv s^−1^ Bq^−1^ m^3^	Sv s^−1^ Bq^−1^ m^3^	Sv s^−1^ Bq^−1^ m^3^
Be-7	4.32E−17	3.18E−17	3.05E−17	2.89E−17	3.19E−15	2.20E−15	2.10E−15	1.99E−15
Na-22	1.79E−15	1.38E−15	1.33E−15	1.27E−15	1.42E−13	1.02E−13	9.80E−14	9.28E−14
K-40	1.21E−16	9.68E−17	9.30E−17	8.97E−17	1.03E−14	7.66E−15	7.35E−15	6.99E−15
K-42	2.2E−16	1.77E−16	1.70E−16	1.64E−16	1.89E−14	1.40E−14	1.34E−14	1.28E−14
Sc-46	1.63E−15	1.26E−15	1.21E−15	1.16E−15	1.32E−13	9.54E−14	9.10E−14	8.63E−14
Cr-51	2.76E−17	2.00E−17	1.91E−17	1.81E−17	1.99E−15	1.37E−15	1.30E−15	1.23E−15
Mn-54	6.87E−16	5.27E−16	5.07E−16	4.85E−16	5.47E−14	3.90E−14	3.72E−14	3.51E−14
Mn-56	1.31E−15	1.03E−15	1.00E−15	9.64E−16	1.11E−13	8.23E−14	7.91E−14	7.50E−14
Fe-59	9.42E−16	7.38E−16	7.10E−16	6.82E−16	7.79E−14	5.70E−14	5.45E−14	5.18E−14
Co-56	2.75E−15	2.19E−15	2.13E−15	2.05E−15	2.38E−13	1.78E−13	1.71E−13	1.63E−13
Co-57	9.39E−17	6.89E−17	6.59E−17	6.43E−17	6.82E−15	4.56E−15	4.33E−15	4.14E−15
Co-58	8.08E−16	6.16E−16	5.92E−16	5.65E−16	6.36E−14	4.51E−14	4.30E−14	4.06E−14
Co-60	1.96E−15	1.55E−15	1.49E−15	1.44E−15	1.64E−13	1.21E−13	1.15E−13	1.10E−13
Ni-65	4.34E−16	3.45E−16	3.32E−16	3.18E−16	3.67E−14	2.70E−14	2.59E−14	2.46E−14
Zn-65	4.66E−16	3.60E−16	3.46E−16	3.32E−16	3.8E−14	2.77E−14	2.64E−14	2.51E−14
Zn-69m	3.6E−16	2.65E−16	2.53E−16	2.40E−16	2.65E−14	1.83E−14	1.74E−14	1.65E−14
Se-75	3.23E−16	2.35E−16	2.24E−16	2.15E−16	2.32E−14	1.59E−14	1.51E−14	1.43E−14
Br-84	1.28E−15	1.04E−15	1.01E−15	9.79E−16	1.16E−13	8.84E−14	8.54E−14	8.16E−14
Rb-86	7.77E−17	5.95E−17	5.72E−17	5.48E−17	6.28E−15	4.53E−15	4.31E−15	4.10E−15
Sr-92	1.03E−15	8.23E−16	7.92E−16	7.63E−16	8.73E−14	6.46E−14	6.19E−14	5.88E−14
Y-90m	5.44E−16	4.00E−16	3.83E−16	3.64E−16	3.99E−14	2.74E−14	2.61E−14	2.47E−14
Y-91	4.39E−18	3.15E−18	3.03E−18	2.90E−18	3.32E−16	2.17E−16	2.08E−16	1.96E−16
Y-91m	4.58E−16	3.37E−16	3.23E−16	3.08E−16	3.41E−14	2.37E−14	2.27E−14	2.13E−14
Y-92	2.14E−16	1.65E−16	1.59E−16	1.53E−16	1.72E−14	1.23E−14	1.18E−14	1.12E−14
Y-93	8.26E−17	6.35E−17	6.13E−17	5.88E−17	6.69E−15	4.84E−15	4.64E−15	4.41E−15
Zr-95	6.14E−16	4.64E−16	4.45E−16	4.25E−16	4.78E−14	3.38E−14	3.22E−14	3.03E−14
Zr-97	7.35E−16	5.57E−16	5.35E−16	5.11E−16	5.76E−14	4.08E−14	3.90E−14	3.67E−14
Nb-93m	2.47E−19	3.89E−21	2.52E−21	1.34E−22	1.74E−16	3.87E−18	2.67E−18	1.90E−19
Nb-95	6.37E−16	4.84E−16	4.65E−16	4.44E−16	5.00E−14	3.54E−14	3.38E−14	3.18E−14
Nb-95m	5.43E−17	3.88E−17	3.70E−17	3.53E−17	4.47E−15	2.62E−15	2.48E−15	2.35E−15
Nb-97	5.7E−16	4.23E−16	4.07E−16	3.88E−16	4.33E−14	3.04E−14	2.90E−14	2.73E−14
Mo-93	1.38E−18	2.17E−20	1.40E−20	6.88E−22	9.76E−16	2.17E−17	1.50E−17	1.06E−18
Mo-99	1.25E−16	9.33E−17	8.95E−17	8.56E−17	9.64E−15	6.72E−15	6.41E−15	6.04E−15
Tc-99m	1E−16	7.33E−17	7.00E−17	6.81E−17	7.31E−15	4.86E−15	4.61E−15	4.41E−15
Ru-103	4.3E−16	3.16E−16	3.03E−16	2.88E−16	3.19E−14	2.20E−14	2.10E−14	1.98E−14
Ru-105	6.34E−16	4.73E−16	4.54E−16	4.33E−16	4.85E−14	3.39E−14	3.22E−14	3.04E−14
Rh-103m	6.26E−19	2.59E−20	2.74E−20	7.72E−21	1.3E−16	5.99E−18	6.30E−18	1.30E−18
Rh-105	6.73E−17	4.87E−17	4.65E−17	4.42E−17	4.85E−15	3.33E−15	3.16E−15	3.00E−15
Rh-106	1.86E−16	1.38E−16	1.33E−16	1.26E−16	1.39E−14	9.75E−15	9.32E−15	8.79E−15
Ag-110m	2.26E−15	1.73E−15	1.67E−15	1.60E−15	1.8E−13	1.29E−13	1.24E−13	1.17E−13
Ag-111	2.36E−17	1.71E−17	1.63E−17	1.55E−17	1.71E−15	1.16E−15	1.10E−15	1.05E−15
Sn-117m	1.26E−16	8.46E−17	8.10E−17	7.77E−17	9.54E−15	5.68E−15	5.41E−15	5.09E−15
Sn-126	4.15E−17	2.46E−17	2.36E−17	2.26E−17	3.31E−15	1.61E−15	1.57E−15	1.43E−15
Sb-124	1.47E−15	1.14E−15	1.10E−15	1.06E−15	1.21E−13	8.85E−14	8.48E−14	8.04E−14
Sb-125	3.76E−16	2.69E−16	2.58E−16	2.45E−16	2.82E−14	1.89E−14	1.81E−14	1.70E−14
Sb-126	2.34E−15	1.75E−15	1.68E−15	1.60E−15	1.79E−13	1.26E−13	1.20E−13	1.13E−13
Sb-127	5.91E−16	4.39E−16	4.21E−16	4.01E−16	4.49E−14	3.14E−14	2.99E−14	2.82E−14
Sb-128	2.6E−15	1.96E−15	1.88E−15	1.79E−15	2.01E−13	1.42E−13	1.35E−13	1.28E−13
Sb-129	1.18E−15	9.12E−16	8.78E−16	8.42E−16	9.56E−14	6.90E−14	6.59E−14	6.24E−14
Sb-130	2.69E−15	2.05E−15	1.97E−15	1.89E−15	2.13E−13	1.51E−13	1.44E−13	1.37E−13
Te-123m	1.19E−16	8.08E−17	7.75E−17	7.42E−17	8.82E−15	5.42E−15	5.16E−15	4.86E−15
Te-125m	2.8E−17	3.14E−18	3.69E−18	1.63E−18	2.64E−15	3.28E−16	3.82E−16	1.76E−16
Te-127m	8.65E−18	1.04E−18	1.20E−18	5.88E−19	8.21E−16	1.06E−16	1.22E−16	5.93E−17
Te-129	5.44E−17	3.78E−17	3.63E−17	3.42E−17	4.14E−15	2.66E−15	2.54E−15	2.38E−15
Te-129m	3.18E−17	1.97E−17	1.91E−17	1.79E−17	2.55E−15	1.44E−15	1.39E−15	1.27E−15
Te-131m	1.19E−15	9.05E−16	8.71E−16	8.33E−16	9.48E−14	6.76E−14	6.45E−14	6.11E−14
Te-132	1.98E−16	1.31E−16	1.26E−16	1.19E−16	1.45E−14	8.87E−15	8.48E−15	7.93E−15
Te-133m	1.51E−15	1.16E−15	1.11E−15	1.07E−15	1.21E−13	8.72E−14	8.33E−14	7.89E−14
Te-134	7.36E−16	5.43E−16	5.21E−16	4.96E−16	5.58E−14	3.85E−14	3.67E−14	3.46E−14
I-129	2.11E−17	3.12E−18	3.60E−18	1.89E−18	1.76E−15	2.77E−16	3.17E−16	1.70E−16
I-130	1.82E−15	1.36E−15	1.30E−15	1.24E−15	1.39E−13	9.75E−14	9.31E−14	8.78E−14
I-131	3.31E−16	2.41E−16	2.31E−16	2.19E−16	2.43E−14	1.67E−14	1.59E−14	1.50E−14
I-132	1.88E−15	1.43E−15	1.37E−15	1.31E−15	1.48E−13	1.05E−13	1.00E−13	9.49E−14
I-133	5.24E−16	3.90E−16	3.75E−16	3.57E−16	3.96E−14	2.77E−14	2.65E−14	2.50E−14
I-134	2.12E−15	1.63E−15	1.57E−15	1.50E−15	1.7E−13	1.22E−13	1.17E−13	1.10E−13
I-135	1.23E−15	9.72E−16	9.37E−16	9.01E−16	1.04E−13	7.64E−14	7.32E−14	6.96E−14
Xe-123	5.25E−16	3.85E−16	3.71E−16	3.54E−16	4.11E−14	2.83E−14	2.71E−14	2.56E−14
Xe-125	2.28E−16	1.51E−16	1.45E−16	1.37E−16	1.71E−14	1.04E−14	9.98E−15	9.31E−15
Xe-127	2.36E−16	1.59E−16	1.52E−16	1.44E−16	1.73E−14	1.08E−14	1.03E−14	9.63E−15
Xe-133	3.69E−17	1.80E−17	1.75E−17	1.60E−17	2.84E−15	1.20E−15	1.19E−15	1.03E−15
Xe-135m	3.68E−16	2.69E−16	2.58E−16	2.46E−16	2.74E−14	1.88E−14	1.81E−14	1.70E−14
Xe-135	2.15E−16	1.54E−16	1.47E−16	1.40E−16	1.53E−14	1.05E−14	9.94E−15	9.43E−15
Xe-138	8.56E−16	6.75E−16	6.55E−16	6.30E−16	7.33E−14	5.45E−14	5.25E−14	5.00E−14
Cs-134	1.31E−15	9.85E−16	9.47E−16	9.03E−16	1.01E−13	7.15E−14	6.82E−14	6.43E−14
Cs-134m	2.12E−17	1.05E−17	1.03E−17	9.38E−18	1.61E−15	7.24E−16	7.10E−16	6.25E−16
Cs-136	1.74E−15	1.33E−15	1.28E−15	1.23E−15	1.39E−13	9.94E−14	9.48E−14	8.98E−14
Cs-137	2.25E−19	1.18E−19	1.15E−19	1.06E−19	1.72E−17	6.32E−18	6.16E−18	5.45E−18
Cs-138	1.81E−15	1.45E−15	1.40E−15	1.34E−15	1.54E−13	1.15E−13	1.10E−13	1.05E−13
Ba-137m	5.11E−16	3.78E−16	3.63E−16	3.46E−16	3.88E−14	2.71E−14	2.59E−14	2.44E−14
Ba-139	4.13E−17	2.94E−17	2.81E−17	2.70E−17	2.98E−15	1.97E−15	1.87E−15	1.77E−15
Ba-140	1.58E−16	1.13E−16	1.08E−16	1.03E−16	1.16E−14	7.88E−15	7.54E−15	7.07E−15
La-140	1.78E−15	1.41E−15	1.36E−15	1.31E−15	1.51E−13	1.11E−13	1.07E−13	1.01E−13
La-141	2.53E−17	1.97E−17	1.89E−17	1.83E−17	2.07E−15	1.48E−15	1.43E−15	1.35E−15
La-142	1.73E−15	1.40E−15	1.36E−15	1.32E−15	1.56E−13	1.19E−13	1.15E−13	1.09E−13
Ce-141	6.22E−17	4.28E−17	4.11E−17	3.94E−17	4.47E−15	2.84E−15	2.70E−15	2.56E−15
Ce-143	2.41E−16	1.66E−16	1.59E−16	1.50E−16	1.77E−14	1.15E−14	1.10E−14	1.03E−14
Ce-144	1.55E−17	9.89E−18	9.55E−18	9.06E−18	1.12E−15	6.57E−16	6.30E−16	5.87E−16
Pr-145	1.78E−17	1.31E−17	1.26E−17	1.20E−17	1.37E−15	9.40E−16	8.98E−16	8.46E−16
Nd-147	1.19E−16	7.97E−17	7.68E−17	7.24E−17	8.72E−15	5.48E−15	5.28E−15	4.90E−15
Pm-148	4.6E−16	3.59E−16	3.45E−16	3.31E−16	3.77E−14	2.74E−14	2.62E−14	2.48E−14
Pm-148m	1.7E−15	1.26E−15	1.21E−15	1.16E−15	1.29E−13	9.06E−14	8.64E−14	8.15E−14
Pm-149	1.09E−17	7.85E−18	7.52E−18	7.14E−18	7.98E−16	5.37E−16	5.10E−16	4.83E−16
Pm-151	2.8E−16	2.01E−16	1.93E−16	1.83E−16	2.06E−14	1.40E−14	1.33E−14	1.26E−14
Eu-152	9.49E−16	7.20E−16	6.93E−16	6.63E−16	7.61E−14	5.42E−14	5.17E−14	4.90E−14
Eu-152m	2.45E−16	1.83E−16	1.76E−16	1.69E−16	1.94E−14	1.36E−14	1.30E−14	1.23E−14
Eu-154	1.01E−15	7.75E−16	7.47E−16	7.17E−16	8.12E−14	5.84E−14	5.57E−14	5.28E−14
Eu-155	4.65E−17	2.99E−17	2.88E−17	2.76E−17	3.38E−15	1.94E−15	1.87E−15	1.74E−15
Eu-156	9.56E−16	7.50E−16	7.27E−16	6.99E−16	8.1E−14	5.99E−14	5.75E−14	5.47E−14
Hf-181	4.53E−16	3.31E−16	3.17E−16	3.01E−16	3.33E−14	2.27E−14	2.17E−14	2.05E−14
Ta-182	1.02E−15	7.90E−16	7.61E−16	7.31E−16	8.35E−14	6.03E−14	5.75E−14	5.47E−14
W-187	3.81E−16	2.80E−16	2.68E−16	2.56E−16	2.86E−14	1.98E−14	1.89E−14	1.78E−14
Pb-210	1.68E−18	6.44E−19	6.61E−19	5.46E−19	1.43E−16	4.10E−17	4.21E−17	3.35E−17
Pb-212	1.19E−16	8.56E−17	8.17E−17	7.82E−17	8.59E−15	5.72E−15	5.43E−15	5.16E−15
Bi-212	8.53E−17	6.53E−17	6.28E−17	6.01E−17	6.85E−15	4.90E−15	4.68E−15	4.43E−15
Ra-224	8.82E−18	6.38E−18	6.09E−18	5.81E−18	6.3E−16	4.30E−16	4.08E−16	3.87E−16
Ra-226	6E−18	4.37E−18	4.18E−18	4.01E−18	4.31E−16	2.90E−16	2.76E−16	2.63E−16
Ac-228	7.02E−16	5.39E−16	5.19E−16	4.98E−16	5.65E−14	4.03E−14	3.84E−14	3.64E−14
Th-228	1.65E−18	1.13E−18	1.08E−18	1.05E−18	1.92E−16	7.55E−17	7.15E−17	6.71E−17
Th-231	1.15E−17	5.88E−18	5.64E−18	5.35E−18	1.43E−15	4.05E−16	3.91E−16	3.43E−16
Th-232	2.12E−19	9.64E−20	9.33E−20	8.83E−20	7.14E−17	7.19E−18	6.54E−18	5.45E−18
Th-234	6.7E−18	4.40E−18	4.22E−18	4.08E−18	5.6E−16	2.84E−16	2.73E−16	2.55E−16
Pa-233	1.82E−16	1.31E−16	1.25E−16	1.19E−16	1.35E−14	8.90E−15	8.45E−15	8.02E−15
U-232	3.41E−19	1.22E−19	1.18E−19	1.10E−19	1.22E−16	1.01E−17	9.15E−18	7.07E−18
U-234	2.36E−19	6.02E−20	5.81E−20	5.29E−20	1.06E−16	5.89E−18	5.13E−18	3.43E−18
U-235	1.33E−16	9.67E−17	9.24E−17	8.89E−17	9.76E−15	6.43E−15	6.11E−15	5.82E−15
U-236	1.77E−19	2.99E−20	2.89E−20	2.51E−20	9.32E−17	3.75E−18	3.11E−18	1.67E−18
U-237	1.06E−16	7.32E−17	7.02E−17	6.75E−17	8.14E−15	4.84E−15	4.62E−15	4.35E−15
U-238	1.44E−19	2.57E−20	2.48E−20	2.17E−20	7.52E−17	3.19E−18	2.68E−18	1.51E−18
Np-237	2.01E−17	1.16E−17	1.12E−17	1.06E−17	1.95E−15	7.80E−16	7.53E−16	6.83E−16
Np-238	4.77E−16	3.65E−16	3.52E−16	3.38E−16	3.86E−14	2.76E−14	2.63E−14	2.49E−14
Np-239	1.42E−16	1.02E−16	9.74E−17	9.39E−17	1.07E−14	6.82E−15	6.48E−15	6.16E−15
Pu-236	2.73E−19	2.55E−20	2.46E−20	1.88E−20	1.14E−16	4.29E−18	3.70E−18	1.40E−18
Pu-238	2.36E−19	1.47E−20	1.40E−20	9.28E−21	1.04E−16	3.37E−18	2.84E−18	7.87E−19
Pu-239	1.48E−19	3.95E−20	3.79E−20	3.43E−20	4.68E−17	3.64E−18	3.33E−18	2.33E−18
Pu-240	2.26E−19	1.56E−20	1.50E−20	1.03E−20	9.8E−17	3.29E−18	2.80E−18	8.43E−19
Pu-242	2.49E−19	5.75E−20	5.56E−20	5.00E−20	8.87E−17	6.43E−18	5.87E−18	4.03E−18
Am-241	1.8E−17	9.28E−18	9.12E−18	8.41E−18	1.57E−15	5.93E−16	5.85E−16	5.12E−16
Am-242	1.11E−17	7.29E−18	6.97E−18	6.79E−18	1.02E−15	4.86E−16	4.63E−16	4.33E−16
Am-242 m	1.16E−18	1.67E−19	1.62E−19	1.32E−19	2.8E−16	1.86E−17	1.80E−17	9.49E−18
Am-243	4.19E−17	2.65E−17	2.55E−17	2.46E−17	3.16E−15	1.69E−15	1.63E−15	1.51E−15
Cm-242	3.21E−19	1.64E−20	1.60E−20	8.42E−21	1.01E−16	3.82E−18	3.55E−18	8.78E−19
Cm-243	1.04E−16	7.43E−17	7.10E−17	6.83E−17	7.84E−15	4.98E−15	4.73E−15	4.49E−15
Cm-244	2.85E−19	2.20E−20	2.14E−20	1.47E−20	8.74E−17	3.94E−18	3.69E−18	1.37E−18
Cm-245	7.7E−17	5.51E−17	5.27E−17	5.14E−17	5.99E−15	3.64E−15	3.46E−15	3.30E−15
Cm-247	2.71E−16	1.99E−16	1.90E−16	1.80E−16	1.99E−14	1.37E−14	1.30E−14	1.23E−14

Full list for all radionuclides can be found at the electronic supplement

Tables 7 and 8 give nuclide-specific equivalent dose rate coefficients for organs of the fetus, for selected radionuclides for soil contamination simulated as a planar source at a depth of 0.5 g cm^−2^ and submersion to contaminated air, respectively. Tables for all radionuclides can be found in the electronic supplement. Moreover, the electronic supplement provides nuclide-specific equivalent dose coefficients for the organs of the mother, i.e., the pregnant model “Katja”. The tabulation of the electronic supplement includes separately the contribution of the primary photons and bremsstrahlung photons generated by electron interactions in soil. As already mentioned, Tables 6, 7, 8 and those of the supplement do not include the contributions of the progeny of the radionuclides produced after deposition or release in the atmosphere.

**Table 7:** Nuclide-specific equivalent dose rate coeffcients of organs of the fetus in Sv s^−1^ Bq^−1^ m^2^, for ground contamination from a planar source at a depth of 0.5 g cm^−2^

Nuclide	Skin	Eyes	Lungs	Heart	Kidneys	Liver	Stomach	Brain	Skeleton
Be-7	2.90E−17	2.87E−17	2.92E−17	2.83E−17	2.97E−17	2.94E−17	2.77E−17	2.87E−17	3.02E−17
Na-22	1.28E−15	1.30E−15	1.28E−15	1.27E−15	1.28E−15	1.29E−15	1.21E−15	1.26E−15	1.30E−15
K-40	9.04E−17	8.82E−17	8.98E−17	8.79E−17	9.04E−17	8.97E−17	8.91E−17	8.89E−17	8.96E−17
K-42	1.65E−16	1.58E−16	1.64E−16	1.59E−16	1.66E−16	1.64E−16	1.64E−16	1.62E−16	1.63E−16
Sc-46	1.17E−15	1.13E−15	1.17E−15	1.14E−15	1.17E−15	1.17E−15	1.14E−15	1.15E−15	1.18E−15
Cr-51	1.83E−17	1.74E−17	1.83E−17	1.77E−17	1.84E−17	1.83E−17	1.78E−17	1.80E−17	1.99E−17
Mn-54	4.91E−16	4.70E−16	4.87E−16	4.89E−16	4.95E−16	4.92E−16	4.80E−16	4.79E−16	4.93E−16
Mn-56	9.70E−16	9.21E−16	9.60E−16	9.57E−16	9.78E−16	9.76E−16	9.50E−16	9.54E−16	9.65E−16
Fe-59	6.88E−16	6.85E−16	6.83E−16	6.74E−16	6.83E−16	6.87E−16	6.58E−16	6.76E−16	6.90E−16
Co-56	2.06E−15	2.03E−15	2.06E−15	2.05E−15	2.07E−15	2.07E−15	1.98E−15	2.04E−15	2.06E−15
Co-57	6.39E−17	6.08E−17	6.42E−17	6.30E−17	6.60E−17	6.53E−17	5.91E−17	6.33E−17	8.41E−17
Co-58	5.71E−16	5.47E−16	5.66E−16	5.67E−16	5.78E−16	5.74E−16	5.59E−16	5.58E−16	5.76E−16
Co-60	1.45E−15	1.47E−15	1.44E−15	1.44E−15	1.44E−15	1.45E−15	1.38E−15	1.42E−15	1.45E−15
Ni-65	3.21E−16	3.10E−16	3.18E−16	3.10E−16	3.20E−16	3.18E−16	3.16E−16	3.15E−16	3.20E−16
Zn-65	3.35E−16	3.21E−16	3.32E−16	3.21E−16	3.32E−16	3.33E−16	3.26E−16	3.29E−16	3.35E−16
Zn-69m	2.42E−16	2.39E−16	2.43E−16	2.38E−16	2.48E−16	2.43E−16	2.28E−16	2.38E−16	2.52E−16
Se-75	2.15E−16	2.04E−16	2.16E−16	2.09E−16	2.18E−16	2.17E−16	2.07E−16	2.13E−16	2.49E−16
Br-84	9.82E−16	9.52E−16	9.80E−16	9.73E−16	9.90E−16	9.87E−16	9.59E−16	9.73E−16	9.78E−16
Rb-86	5.53E−17	5.27E−17	5.48E−17	5.30E−17	5.48E−17	5.48E−17	5.39E−17	5.44E−17	5.57E−17
Sr-92	7.69E−16	7.73E−16	7.66E−16	7.61E−16	7.68E−16	7.69E−16	7.40E−16	7.56E−16	7.68E−16
Y-90m	3.65E−16	3.54E−16	3.68E−16	3.55E−16	3.73E−16	3.69E−16	3.51E−16	3.62E−16	3.94E−16
Y-91	2.92E−18	2.87E−18	2.91E−18	2.87E−18	2.94E−18	2.93E−18	2.74E−18	2.87E−18	3.18E−18
Y-91m	3.10E−16	2.94E−16	3.09E−16	3.03E−16	3.16E−16	3.11E−16	2.99E−16	3.05E−16	3.20E−16
Y-92	1.54E−16	1.51E−16	1.54E−16	1.52E−16	1.54E−16	1.53E−16	1.48E−16	1.51E−16	1.56E−16
Y-93	5.91E−17	5.63E−17	5.88E−17	5.77E−17	5.94E−17	5.93E−17	5.75E−17	5.83E−17	6.09E−17
Zr-95	4.30E−16	4.08E−16	4.26E−16	4.28E−16	4.36E−16	4.33E−16	4.22E−16	4.19E−16	4.34E−16
Zr-97	5.17E−16	4.92E−16	5.12E−16	5.13E−16	5.23E−16	5.20E−16	5.05E−16	5.05E−16	5.22E−16
Nb-93m	2.50E−22	9.78E−24	6.90E−23	1.18E−23	9.64E−23	7.67E−23	7.27E−24	1.69E−22	2.72E−22
Nb-95	4.49E−16	4.27E−16	4.44E−16	4.48E−16	4.55E−16	4.52E−16	4.42E−16	4.37E−16	4.52E−16
Nb-95m	3.54E−17	3.27E−17	3.56E−17	3.40E−17	3.58E−17	3.55E−17	3.48E−17	3.53E−17	4.03E−17
Nb-97	3.92E−16	3.70E−16	3.89E−16	3.87E−16	3.98E−16	3.93E−16	3.81E−16	3.83E−16	3.99E−16
Mo-93	1.33E−21	1.41E−23	3.24E−22	1.99E−23	4.74E−22	3.65E−22	8.27E−24	8.79E−22	1.41E−21
Mo-99	8.64E−17	8.21E−17	8.58E−17	8.59E−17	8.76E−17	8.71E−17	8.43E−17	8.45E−17	8.95E−17
Tc-99m	6.76E−17	6.58E−17	6.80E−17	6.71E−17	6.92E−17	6.90E−17	6.25E−17	6.73E−17	8.66E−17
Ru-103	2.90E−16	2.83E−16	2.91E−16	2.82E−16	2.96E−16	2.93E−16	2.78E−16	2.86E−16	3.00E−16
Ru-105	4.37E−16	4.17E−16	4.35E−16	4.31E−16	4.43E−16	4.39E−16	4.23E−16	4.27E−16	4.49E−16
Rh-103m	9.37E−21	4.50E−21	7.20E−21	5.48E−21	7.94E−21	7.58E−21	4.14E−21	8.27E−21	1.38E−20
Rh-105	4.45E−17	4.22E−17	4.45E−17	4.31E−17	4.48E−17	4.44E−17	4.34E−17	4.38E−17	4.87E−17
Rh-106	1.26E−16	1.22E−16	1.26E−16	1.24E−16	1.28E−16	1.27E−16	1.22E−16	1.25E−16	1.31E−16
Ag-110m	1.61E−15	1.56E−15	1.61E−15	1.59E−15	1.63E−15	1.61E−15	1.57E−15	1.58E−15	1.63E−15
Ag-111	1.56E−17	1.49E−17	1.56E−17	1.52E−17	1.58E−17	1.56E−17	1.49E−17	1.54E−17	1.70E−17
Sn-117m	7.72E−17	7.47E−17	7.77E−17	7.62E−17	7.88E−17	7.86E−17	7.20E−17	7.72E−17	9.62E−17
Sn-126	2.26E−17	1.99E−17	2.25E−17	2.16E−17	2.34E−17	2.29E−17	1.95E−17	2.22E−17	3.29E−17
Sb-124	1.06E−15	1.00E−15	1.05E−15	1.04E−15	1.07E−15	1.07E−15	1.04E−15	1.04E−15	1.06E−15
Sb-125	2.47E−16	2.37E−16	2.47E−16	2.43E−16	2.52E−16	2.48E−16	2.36E−16	2.42E−16	2.57E−16
Sb-126	1.61E−15	1.54E−15	1.61E−15	1.60E−15	1.64E−15	1.62E−15	1.57E−15	1.58E−15	1.65E−15
Sb-127	4.05E−16	3.88E−16	4.03E−16	4.00E−16	4.11E−16	4.08E−16	3.93E−16	3.96E−16	4.15E−16
Sb-128	1.81E−15	1.72E−15	1.80E−15	1.79E−15	1.83E−15	1.82E−15	1.77E−15	1.77E−15	1.85E−15
Sb-129	8.49E−16	8.15E−16	8.44E−16	8.36E−16	8.55E−16	8.49E−16	8.27E−16	8.33E−16	8.55E−16
Sb-130	1.90E−15	1.83E−15	1.89E−15	1.88E−15	1.92E−15	1.91E−15	1.86E−15	1.87E−15	1.94E−15
Te-123m	7.38E−17	7.13E−17	7.43E−17	7.27E−17	7.52E−17	7.51E−17	6.88E−17	7.37E−17	9.19E−17
Te-125m	1.87E−18	1.08E−18	1.61E−18	1.23E−18	1.72E−18	1.66E−18	8.85E−19	1.73E−18	2.86E−18
Te-127m	6.58E−19	4.16E−19	5.79E−19	4.70E−19	6.17E−19	5.96E−19	3.64E−19	6.17E−19	9.84E−19
Te-129	3.44E−17	3.36E−17	3.45E−17	3.35E−17	3.50E−17	3.46E−17	3.29E−17	3.39E−17	3.60E−17
Te-129m	1.81E−17	1.70E−17	1.79E−17	1.78E−17	1.83E−17	1.82E−17	1.75E−17	1.76E−17	1.86E−17
Te-131m	8.41E−16	8.09E−16	8.35E−16	8.31E−16	8.47E−16	8.44E−16	8.18E−16	8.24E−16	8.55E−16
Te-132	1.19E−16	1.10E−16	1.20E−16	1.14E−16	1.21E−16	1.20E−16	1.16E−16	1.19E−16	1.38E−16
Te-133m	1.08E−15	1.04E−15	1.07E−15	1.06E−15	1.08E−15	1.08E−15	1.04E−15	1.06E−15	1.09E−15
Te-134	5.00E−16	4.76E−16	4.99E−16	4.93E−16	5.08E−16	5.03E−16	4.85E−16	4.91E−16	5.26E−16
I-129	2.10E−18	1.27E−18	1.90E−18	1.48E−18	2.00E−18	1.96E−18	1.10E−18	1.94E−18	3.30E−18
I-130	1.25E−15	1.20E−15	1.24E−15	1.24E−15	1.27E−15	1.26E−15	1.21E−15	1.23E−15	1.28E−15
I-131	2.21E−16	2.14E−16	2.21E−16	2.17E−16	2.25E−16	2.21E−16	2.10E−16	2.16E−16	2.35E−16
I-132	1.32E−15	1.27E−15	1.32E−15	1.31E−15	1.34E−15	1.33E−15	1.29E−15	1.30E−15	1.34E−15
I-133	3.59E−16	3.47E−16	3.58E−16	3.52E−16	3.65E−16	3.61E−16	3.44E−16	3.54E−16	3.70E−16
I-134	1.51E−15	1.46E−15	1.51E−15	1.49E−15	1.52E−15	1.51E−15	1.47E−15	1.48E−15	1.53E−15
I-135	9.07E−16	8.88E−16	9.01E−16	8.89E−16	9.07E−16	9.08E−16	8.79E−16	8.93E−16	9.07E−16
Xe-123	3.56E−16	3.42E−16	3.54E−16	3.47E−16	3.60E−16	3.57E−16	3.42E−16	3.52E−16	3.75E−16
Xe-125	1.37E−16	1.29E−16	1.38E−16	1.33E−16	1.39E−16	1.38E−16	1.32E−16	1.36E−16	1.56E−16
Xe-127	1.44E−16	1.36E−16	1.45E−16	1.40E−16	1.47E−16	1.45E−16	1.38E−16	1.44E−16	1.67E−16
Xe-133	1.62E−17	1.39E−17	1.60E−17	1.51E−17	1.66E−17	1.63E−17	1.33E−17	1.59E−17	2.40E−17
Xe-135m	2.47E−16	2.38E−16	2.46E−16	2.40E−16	2.52E−16	2.48E−16	2.37E−16	2.44E−16	2.56E−16
Xe-135	1.41E−16	1.31E−16	1.41E−16	1.35E−16	1.42E−16	1.41E−16	1.39E−16	1.40E−16	1.58E−16
Xe-138	6.32E−16	5.97E−16	6.25E−16	6.17E−16	6.38E−16	6.38E−16	6.18E−16	6.25E−16	6.35E−16
Cs-134	9.13E−16	8.65E−16	9.06E−16	9.04E−16	9.25E−16	9.17E−16	8.90E−16	8.92E−16	9.26E−16
Cs-134m	9.41E−18	8.72E−18	9.37E−18	9.05E−18	9.64E−18	9.53E−18	8.33E−18	9.27E−18	1.26E−17
Cs-136	1.24E−15	1.20E−15	1.23E−15	1.22E−15	1.24E−15	1.24E−15	1.20E−15	1.21E−15	1.26E−15
Cs-137	1.06E−19	9.48E−20	1.06E−19	1.02E−19	1.09E−19	1.08E−19	9.47E−20	1.04E−19	1.45E−19
Cs-138	1.34E−15	1.31E−15	1.34E−15	1.32E−15	1.35E−15	1.34E−15	1.30E−15	1.33E−15	1.35E−15
Ba-137m	3.49E−16	3.29E−16	3.46E−16	3.45E−16	3.55E−16	3.51E−16	3.40E−16	3.41E−16	3.56E−16
Ba-139	2.69E−17	2.59E−17	2.71E−17	2.65E−17	2.75E−17	2.73E−17	2.54E−17	2.69E−17	3.25E−17
Ba-140	1.03E−16	9.93E−17	1.03E−16	1.01E−16	1.05E−16	1.04E−16	9.90E−17	1.02E−16	1.09E−16
La-140	1.32E−15	1.25E−15	1.31E−15	1.28E−15	1.32E−15	1.31E−15	1.30E−15	1.30E−15	1.31E−15
La-141	1.84E−17	1.83E−17	1.83E−17	1.82E−17	1.83E−17	1.84E−17	1.75E−17	1.80E−17	1.88E−17
La-142	1.32E−15	1.27E−15	1.31E−15	1.30E−15	1.33E−15	1.32E−15	1.28E−15	1.31E−15	1.31E−15
Ce-141	3.92E−17	3.78E−17	3.94E−17	3.87E−17	4.01E−17	4.00E−17	3.59E−17	3.90E−17	5.01E−17
Ce-143	1.51E−16	1.41E−16	1.51E−16	1.46E−16	1.53E−16	1.52E−16	1.45E−16	1.49E−16	1.65E−16
Ce-144	9.04E−18	8.53E−18	9.06E−18	8.83E−18	9.28E−18	9.21E−18	8.15E−18	8.94E−18	1.20E−17
Pr-145	1.21E−17	1.16E−17	1.21E−17	1.19E−17	1.22E−17	1.21E−17	1.17E−17	1.19E−17	1.26E−17
Nd-147	7.29E−17	6.82E−17	7.27E−17	7.05E−17	7.45E−17	7.33E−17	6.80E−17	7.16E−17	8.29E−17
Pm-148	3.34E−16	3.24E−16	3.33E−16	3.26E−16	3.36E−16	3.32E−16	3.26E−16	3.28E−16	3.35E−16
Pm-148 m	1.17E−15	1.11E−15	1.16E−15	1.15E−15	1.18E−15	1.17E−15	1.13E−15	1.14E−15	1.20E−15
Pm-149	7.19E−18	6.76E−18	7.18E−18	6.97E−18	7.24E−18	7.20E−18	7.03E−18	7.10E−18	7.92E−18
Pm-151	1.84E−16	1.75E−16	1.84E−16	1.80E−16	1.87E−16	1.85E−16	1.77E−16	1.81E−16	2.01E−16
Eu-152	6.68E−16	6.49E−16	6.66E−16	6.53E−16	6.70E−16	6.66E−16	6.44E−16	6.56E−16	6.86E−16
Eu-152m	1.70E−16	1.65E−16	1.70E−16	1.68E−16	1.71E−16	1.70E−16	1.63E−16	1.67E−16	1.75E−16
Eu-154	7.22E−16	7.17E−16	7.21E−16	7.17E−16	7.24E−16	7.23E−16	6.88E−16	7.09E−16	7.40E−16
Eu-155	2.76E−17	2.45E−17	2.76E−17	2.65E−17	2.87E−17	2.81E−17	2.41E−17	2.71E−17	3.96E−17
Eu-156	7.03E−16	6.81E−16	6.96E−16	6.93E−16	7.05E−16	7.08E−16	6.79E−16	6.93E−16	7.03E−16
Hf-181	3.02E−16	2.95E−16	3.04E−16	2.95E−16	3.09E−16	3.06E−16	2.87E−16	2.99E−16	3.28E−16
Ta-182	7.36E−16	7.32E−16	7.33E−16	7.23E−16	7.34E−16	7.38E−16	7.01E−16	7.24E−16	7.59E−16
W-187	2.58E−16	2.45E−16	2.57E−16	2.54E−16	2.62E−16	2.60E−16	2.49E−16	2.53E−16	2.71E−16
Pb-210	5.58E−19	4.30E−19	5.55E−19	5.15E−19	5.83E−19	5.67E−19	4.20E−19	5.27E−19	9.05E−19
Pb-212	7.84E−17	7.20E−17	7.86E−17	7.52E−17	7.96E−17	7.88E−17	7.56E−17	7.78E−17	9.36E−17
Bi-212	6.06E−17	5.76E−17	6.01E−17	5.95E−17	6.12E−17	6.07E−17	5.97E−17	5.94E−17	6.12E−17
Ra-224	5.83E−18	5.39E−18	5.86E−18	5.60E−18	5.90E−18	5.85E−18	5.73E−18	5.80E−18	6.68E−18
Ra-226	4.01E−18	3.75E−18	4.04E−18	3.88E−18	4.09E−18	4.05E−18	3.84E−18	4.00E−18	4.84E−18
Ac-228	5.02E−16	4.85E−16	5.02E−16	4.93E−16	5.04E−16	4.99E−16	4.84E−16	4.93E−16	5.12E−16
Th-228	1.05E−18	9.54E−19	1.05E−18	1.01E−18	1.07E−18	1.06E−18	9.49E−19	1.04E−18	1.39E−18
Th-231	5.36E−18	4.76E−18	5.33E−18	5.12E−18	5.53E−18	5.42E−18	4.63E−18	5.28E−18	7.67E−18
Th-232	8.83E−20	7.65E−20	8.87E−20	8.47E−20	9.19E−20	9.01E−20	7.90E−20	8.64E−20	1.32E−19
Th-234	4.08E−18	3.59E−18	4.08E−18	3.92E−18	4.25E−18	4.15E−18	3.63E−18	4.00E−18	5.94E−18
Pa-233	1.20E−16	1.13E−16	1.20E−16	1.16E−16	1.22E−16	1.20E−16	1.15E−16	1.18E−16	1.37E−16
U-232	1.10E−19	9.88E−20	1.10E−19	1.07E−19	1.14E−19	1.12E−19	9.88E−20	1.08E−19	1.54E−19
U-234	5.31E−20	4.68E−20	5.31E−20	5.13E−20	5.51E−20	5.40E−20	4.63E−20	5.18E−20	7.69E−20
U-235	8.87E−17	8.34E−17	8.94E−17	8.63E−17	9.06E−17	8.99E−17	8.47E−17	8.86E−17	1.08E−16
U-236	2.53E−20	2.17E−20	2.52E−20	2.42E−20	2.63E−20	2.57E−20	2.14E−20	2.45E−20	3.75E−20
U-237	6.75E−17	6.15E−17	6.78E−17	6.52E−17	6.95E−17	6.84E−17	6.31E−17	6.67E−17	8.77E−17
U-238	2.19E−20	1.90E−20	2.17E−20	2.09E−20	2.26E−20	2.21E−20	1.88E−20	2.12E−20	3.06E−20
Np-237	1.06E−17	9.52E−18	1.06E−17	1.02E−17	1.10E−17	1.08E−17	9.33E−18	1.05E−17	1.49E−17
Np-238	3.41E−16	3.32E−16	3.42E−16	3.32E−16	3.40E−16	3.37E−16	3.26E−16	3.35E−16	3.46E−16
Np-239	9.40E−17	8.70E−17	9.42E−17	9.09E−17	9.63E−17	9.49E−17	8.95E−17	9.28E−17	1.16E−16
Pu-236	1.92E−20	1.60E−20	1.88E−20	1.78E−20	1.96E−20	1.91E−20	1.58E−20	1.84E−20	2.77E−20
Pu-238	9.63E−21	7.63E−21	9.25E−21	8.54E−21	9.70E−21	9.47E−21	7.55E−21	9.15E−21	1.41E−20
Pu-239	3.46E−20	3.18E−20	3.45E−20	3.35E−20	3.56E−20	3.48E−20	3.12E−20	3.38E−20	4.39E−20
Pu-240	1.07E−20	8.53E−21	1.03E−20	9.61E−21	1.08E−20	1.06E−20	8.43E−21	1.02E−20	1.57E−20
Pu-242	5.06E−20	4.73E−20	5.01E−20	4.89E−20	5.09E−20	5.05E−20	4.77E−20	4.96E−20	5.43E−20
Am-241	8.45E−18	6.97E−18	8.48E−18	8.09E−18	8.81E−18	8.59E−18	7.32E−18	8.24E−18	1.32E−17
Am-242	6.78E−18	6.22E−18	6.79E−18	6.58E−18	7.05E−18	6.90E−18	6.21E−18	6.65E−18	9.26E−18
Am-242 m	1.34E−19	1.17E−19	1.31E−19	1.26E−19	1.36E−19	1.34E−19	1.16E−19	1.30E−19	1.85E−19
Am-243	2.46E−17	2.14E−17	2.46E−17	2.33E−17	2.56E−17	2.51E−17	2.14E−17	2.41E−17	3.69E−17
Cm-242	9.00E−21	6.78E−21	8.27E−21	7.53E−21	8.70E−21	8.47E−21	6.64E−21	8.47E−21	1.25E−20
Cm-243	6.84E−17	6.33E−17	6.86E−17	6.60E−17	6.99E−17	6.90E−17	6.55E−17	6.77E−17	8.37E−17
Cm-244	1.52E−20	1.32E−20	1.46E−20	1.39E−20	1.50E−20	1.48E−20	1.32E−20	1.47E−20	1.77E−20
Cm-245	5.12E−17	4.74E−17	5.14E−17	4.99E−17	5.31E−17	5.21E−17	4.73E−17	5.05E−17	6.83E−17
Cm-247	1.81E−16	1.78E−16	1.82E−16	1.80E−16	1.86E−16	1.81E−16	1.70E−16	1.78E−16	1.92E−16

Full list for all radionuclides can be found at the electronic supplement

**Table 8:** Nuclide-specific equivalent dose rate coeffcients of organs of the fetus in Sv s^−1^ Bq^−1^ m^3^ for submersion to contaminated air

Nuclide	Skin	Eyes	Lungs	Heart	Kidneys	Liver	Stomach	Brain	Skeleton
Be-7	2.03E−15	1.85E−15	2.01E−15	1.97E−15	2.12E−15	2.11E−15	2.01E−15	1.83E−15	2.11E−15
Na-22	9.38E−14	8.54E−14	9.33E−14	9.24E−14	9.76E−14	9.70E−14	9.38E−14	8.56E−14	9.43E−14
K-40	7.06E−15	6.21E−15	6.95E−15	6.94E−15	7.30E−15	7.21E−15	6.91E−15	6.44E−15	6.89E−15
K-42	1.29E−14	1.13E−14	1.27E−14	1.27E−14	1.34E−14	1.32E−14	1.26E−14	1.17E−14	1.26E−14
Sc-46	8.75E−14	8.10E−14	8.77E−14	8.72E−14	9.00E−14	9.05E−14	8.99E−14	8.01E−14	8.62E−14
Cr-51	1.25E−15	1.07E−15	1.26E−15	1.25E−15	1.33E−15	1.31E−15	1.25E−15	1.12E−15	1.36E−15
Mn-54	3.56E−14	3.31E−14	3.60E−14	3.56E−14	3.70E−14	3.71E−14	3.62E−14	3.25E−14	3.55E−14
Mn-56	7.58E−14	7.06E−14	7.68E−14	7.53E−14	7.95E−14	7.90E−14	7.48E−14	7.00E−14	7.45E−14
Fe-59	5.24E−14	4.77E−14	5.22E−14	5.20E−14	5.39E−14	5.39E−14	5.31E−14	4.80E−14	5.13E−14
Co-56	1.65E−13	1.56E−13	1.66E−13	1.66E−13	1.72E−13	1.70E−13	1.64E−13	1.53E−13	1.62E−13
Co-57	4.20E−15	3.68E−15	4.25E−15	4.13E−15	4.54E−15	4.50E−15	3.84E−15	3.60E−15	5.32E−15
Co-58	4.11E−14	3.81E−14	4.15E−14	4.09E−14	4.30E−14	4.29E−14	4.13E−14	3.75E−14	4.14E−14
Co-60	1.11E−13	1.00E−13	1.10E−13	1.10E−13	1.14E−13	1.14E−13	1.11E−13	1.02E−13	1.09E−13
Ni-65	2.49E−14	2.22E−14	2.46E−14	2.45E−14	2.57E−14	2.55E−14	2.47E−14	2.28E−14	2.44E−14
Zn-65	2.54E−14	2.33E−14	2.53E−14	2.52E−14	2.61E−14	2.62E−14	2.60E−14	2.33E−14	2.49E−14
Zn-69m	1.68E−14	1.52E−14	1.67E−14	1.64E−14	1.76E−14	1.75E−14	1.66E−14	1.51E−14	1.77E−14
Se-75	1.45E−14	1.26E−14	1.47E−14	1.44E−14	1.55E−14	1.53E−14	1.41E−14	1.28E−14	1.66E−14
Br-84	8.23E−14	7.94E−14	8.29E−14	8.38E−14	8.60E−14	8.52E−14	8.11E−14	7.72E−14	8.06E−14
Rb-86	4.16E−15	3.84E−15	4.16E−15	4.14E−15	4.26E−15	4.29E−15	4.28E−15	3.81E−15	4.09E−15
Sr-92	5.95E−14	5.29E−14	5.88E−14	5.86E−14	6.15E−14	6.08E−14	5.88E−14	5.44E−14	5.81E−14
Y-90m	2.52E−14	2.27E−14	2.50E−14	2.45E−14	2.64E−14	2.62E−14	2.45E−14	2.26E−14	2.71E−14
Y-91	1.99E−16	1.77E−16	1.98E−16	1.95E−16	2.08E−16	2.06E−16	1.96E−16	1.78E−16	2.05E−16
Y-91m	2.15E−14	2.00E−14	2.17E−14	2.12E−14	2.27E−14	2.26E−14	2.14E−14	1.96E−14	2.24E−14
Y-92	1.13E−14	1.04E−14	1.13E−14	1.13E−14	1.17E−14	1.17E−14	1.15E−14	1.03E−14	1.12E−14
Y-93	4.46E−15	4.07E−15	4.51E−15	4.41E−15	4.68E−15	4.64E−15	4.39E−15	4.09E−15	4.49E−15
Zr-95	3.06E−14	2.84E−14	3.13E−14	3.07E−14	3.25E−14	3.22E−14	3.02E−14	2.81E−14	3.10E−14
Zr-97	3.71E−14	3.43E−14	3.77E−14	3.71E−14	3.92E−14	3.89E−14	3.67E−14	3.40E−14	3.74E−14
Nb-93m	4.16E−19	2.02E−21	9.78E−20	3.00E−21	2.52E−19	1.77E−19	2.24E−21	1.56E−20	6.63E−20
Nb-95	3.22E−14	2.99E−14	3.27E−14	3.22E−14	3.39E−14	3.37E−14	3.20E−14	2.94E−14	3.24E−14
Nb-95m	2.38E−15	2.07E−15	2.39E−15	2.34E−15	2.54E−15	2.49E−15	2.29E−15	2.11E−15	2.71E−15
Nb-97	2.75E−14	2.54E−14	2.80E−14	2.74E−14	2.93E−14	2.89E−14	2.70E−14	2.51E−14	2.80E−14
Mo-93	2.32E−18	8.09E−21	5.41E−19	1.12E−20	1.40E−18	9.85E−19	8.40E−21	8.42E−20	3.64E−19
Mo-99	6.11E−15	5.62E−15	6.21E−15	6.10E−15	6.46E−15	6.41E−15	6.00E−15	5.55E−15	6.30E−15
Tc-99m	4.46E−15	3.92E−15	4.51E−15	4.39E−15	4.76E−15	4.75E−15	4.17E−15	3.86E−15	5.56E−15
Ru-103	2.01E−14	1.85E−14	2.01E−14	1.97E−14	2.12E−14	2.10E−14	2.00E−14	1.83E−14	2.10E−14
Ru-105	3.07E−14	2.82E−14	3.12E−14	3.06E−14	3.26E−14	3.23E−14	3.03E−14	2.80E−14	3.16E−14
Rh-103m	1.98E−18	4.96E−19	1.20E−18	7.60E−19	2.03E−18	1.66E−18	5.99E−19	4.09E−19	1.08E−18
Rh-105	3.02E−15	2.60E−15	3.07E−15	3.02E−15	3.23E−15	3.18E−15	3.03E−15	2.72E−15	3.31E−15
Rh-106	8.87E−15	8.21E−15	8.93E−15	8.76E−15	9.35E−15	9.29E−15	8.84E−15	8.08E−15	9.16E−15
Ag-110m	1.18E−13	1.09E−13	1.19E−13	1.18E−13	1.23E−13	1.23E−13	1.18E−13	1.08E−13	1.18E−13
Ag-111	1.06E−15	9.22E−16	1.07E−15	1.05E−15	1.13E−15	1.11E−15	1.05E−15	9.49E−16	1.16E−15
Sn-117m	5.17E−15	4.51E−15	5.18E−15	5.05E−15	5.48E−15	5.46E−15	4.84E−15	4.47E−15	6.27E−15
Sn-126	1.46E−15	1.20E−15	1.48E−15	1.42E−15	1.62E−15	1.60E−15	1.30E−15	1.19E−15	1.94E−15
Sb-124	8.12E−14	7.38E−14	8.16E−14	8.00E−14	8.54E−14	8.43E−14	7.91E−14	7.45E−14	8.06E−14
Sb-125	1.72E−14	1.57E−14	1.73E−14	1.70E−14	1.82E−14	1.81E−14	1.69E−14	1.56E−14	1.80E−14
Sb-126	1.14E−13	1.05E−13	1.16E−13	1.14E−13	1.21E−13	1.20E−13	1.12E−13	1.04E−13	1.17E−13
Sb-127	2.85E−14	2.62E−14	2.89E−14	2.83E−14	3.02E−14	2.99E−14	2.81E−14	2.60E−14	2.93E−14
Sb-128	1.29E−13	1.19E−13	1.31E−13	1.29E−13	1.36E−13	1.35E−13	1.28E−13	1.18E−13	1.31E−13
Sb-129	6.32E−14	5.83E−14	6.36E−14	6.28E−14	6.58E−14	6.57E−14	6.34E−14	5.79E−14	6.28E−14
Sb-130	1.38E−13	1.27E−13	1.40E−13	1.38E−13	1.45E−13	1.44E−13	1.39E−13	1.26E−13	1.40E−13
Te-123m	4.94E−15	4.30E−15	4.95E−15	4.82E−15	5.24E−15	5.22E−15	4.62E−15	4.26E−15	5.99E−15
Te-125m	2.14E−16	9.23E−17	1.89E−16	1.56E−16	2.51E−16	2.37E−16	1.16E−16	8.52E−17	1.99E−16
Te-127m	7.10E−17	3.36E−17	6.33E−17	5.34E−17	8.28E−17	7.84E−17	4.10E−17	3.15E−17	6.76E−17
Te-129	2.42E−15	2.20E−15	2.41E−15	2.37E−15	2.54E−15	2.53E−15	2.40E−15	2.18E−15	2.52E−15
Te-129m	1.29E−15	1.17E−15	1.31E−15	1.28E−15	1.38E−15	1.36E−15	1.24E−15	1.16E−15	1.31E−15
Te-131m	6.18E−14	5.69E−14	6.24E−14	6.15E−14	6.46E−14	6.43E−14	6.19E−14	5.65E−14	6.21E−14
Te-132	8.07E−15	6.92E−15	8.07E−15	7.85E−15	8.62E−15	8.45E−15	7.61E−15	7.03E−15	9.24E−15
Te-133m	7.99E−14	7.38E−14	8.05E−14	7.95E−14	8.32E−14	8.31E−14	8.05E−14	7.31E−14	7.98E−14
Te-134	3.50E−14	3.20E−14	3.54E−14	3.47E−14	3.70E−14	3.67E−14	3.45E−14	3.17E−14	3.68E−14
I-129	1.99E−16	9.14E−17	1.85E−16	1.53E−16	2.37E−16	2.27E−16	1.18E−16	9.02E−17	2.06E−16
I-130	8.84E−14	8.19E−14	8.97E−14	8.81E−14	9.37E−14	9.29E−14	8.76E−14	8.08E−14	9.06E−14
I-131	1.52E−14	1.35E−14	1.53E−14	1.51E−14	1.61E−14	1.60E−14	1.51E−14	1.37E−14	1.63E−14
I-132	9.57E−14	8.84E−14	9.70E−14	9.54E−14	1.01E−13	1.00E−13	9.51E−14	8.76E−14	9.65E−14
I-133	2.52E−14	2.34E−14	2.53E−14	2.49E−14	2.65E−14	2.64E−14	2.52E−14	2.29E−14	2.60E−14
I-134	1.12E−13	1.03E−13	1.13E−13	1.11E−13	1.16E−13	1.16E−13	1.13E−13	1.02E−13	1.11E−13
I-135	7.04E−14	6.38E−14	7.02E−14	6.95E−14	7.30E−14	7.25E−14	6.99E−14	6.45E−14	6.89E−14
Xe-123	2.59E−14	2.36E−14	2.60E−14	2.55E−14	2.72E−14	2.70E−14	2.55E−14	2.34E−14	2.69E−14
Xe-125	9.48E−15	8.28E−15	9.47E−15	9.24E−15	1.00E−14	9.92E−15	9.06E−15	8.32E−15	1.06E−14
Xe-127	9.80E−15	8.55E−15	9.78E−15	9.54E−15	1.04E−14	1.03E−14	9.28E−15	8.57E−15	1.12E−14
Xe-133	1.07E−15	8.39E−16	1.08E−15	1.02E−15	1.19E−15	1.18E−15	9.33E−16	8.34E−16	1.41E−15
Xe-135m	1.71E−14	1.59E−14	1.71E−14	1.68E−14	1.80E−14	1.79E−14	1.71E−14	1.55E−14	1.79E−14
Xe-135	9.54E−15	8.26E−15	9.63E−15	9.42E−15	1.02E−14	1.00E−14	9.28E−15	8.50E−15	1.07E−14
Xe-138	5.05E−14	4.64E−14	5.11E−14	4.97E−14	5.32E−14	5.26E−14	4.88E−14	4.66E−14	5.00E−14
Cs-134	6.50E−14	6.02E−14	6.59E−14	6.47E−14	6.84E−14	6.81E−14	6.48E−14	5.94E−14	6.60E−14
Cs-134m	6.45E−16	5.31E−16	6.45E−16	6.17E−16	7.04E−16	6.96E−16	5.66E−16	5.21E−16	7.96E−16
Cs-136	9.10E−14	8.38E−14	9.15E−14	9.07E−14	9.42E−14	9.44E−14	9.27E−14	8.31E−14	9.10E−14
Cs-137	5.62E−18	4.53E−18	5.62E−18	5.40E−18	6.18E−18	6.07E−18	5.01E−18	4.53E−18	6.98E−18
Cs-138	1.06E−13	9.77E−14	1.06E−13	1.05E−13	1.10E−13	1.09E−13	1.05E−13	9.79E−14	1.04E−13
Ba-137m	2.45E−14	2.27E−14	2.50E−14	2.45E−14	2.62E−14	2.59E−14	2.41E−14	2.24E−14	2.50E−14
Ba-139	1.80E−15	1.58E−15	1.80E−15	1.76E−15	1.90E−15	1.89E−15	1.70E−15	1.58E−15	2.11E−15
Ba-140	7.13E−15	6.55E−15	7.17E−15	7.03E−15	7.56E−15	7.50E−15	7.09E−15	6.45E−15	7.58E−15
La-140	1.02E−13	9.19E−14	1.02E−13	1.01E−13	1.07E−13	1.05E−13	1.00E−13	9.37E−14	1.01E−13
La-141	1.37E−15	1.23E−15	1.36E−15	1.34E−15	1.43E−15	1.41E−15	1.34E−15	1.25E−15	1.36E−15
La-142	1.10E−13	1.06E−13	1.12E−13	1.12E−13	1.16E−13	1.15E−13	1.07E−13	1.03E−13	1.08E−13
Ce-141	2.60E−15	2.25E−15	2.61E−15	2.54E−15	2.77E−15	2.76E−15	2.42E−15	2.23E−15	3.21E−15
Ce-143	1.04E−14	9.14E−15	1.06E−14	1.03E−14	1.11E−14	1.10E−14	1.02E−14	9.32E−15	1.12E−14
Ce-144	5.99E−16	5.10E−16	6.04E−16	5.84E−16	6.47E−16	6.43E−16	5.46E−16	5.03E−16	7.51E−16
Pr-145	8.56E−16	7.87E−16	8.65E−16	8.54E−16	8.97E−16	8.95E−16	8.58E−16	7.78E−16	8.71E−16
Nd-147	4.97E−15	4.44E−15	5.01E−15	4.87E−15	5.33E−15	5.28E−15	4.81E−15	4.38E−15	5.50E−15
Pm-148	2.51E−14	2.26E−14	2.50E−14	2.48E−14	2.61E−14	2.59E−14	2.50E−14	2.29E−14	2.49E−14
Pm-148m	8.22E−14	7.62E−14	8.33E−14	8.18E−14	8.67E−14	8.63E−14	8.22E−14	7.51E−14	8.44E−14
Pm-149	4.88E−16	4.25E−16	4.96E−16	4.86E−16	5.20E−16	5.13E−16	4.86E−16	4.37E−16	5.32E−16
Pm-151	1.27E−14	1.13E−14	1.28E−14	1.26E−14	1.35E−14	1.34E−14	1.25E−14	1.14E−14	1.38E−14
Eu-152	4.96E−14	4.49E−14	4.96E−14	4.92E−14	5.15E−14	5.14E−14	4.99E−14	4.51E−14	4.98E−14
Eu-152m	1.25E−14	1.14E−14	1.25E−14	1.24E−14	1.29E−14	1.30E−14	1.27E−14	1.12E−14	1.25E−14
Eu-154	5.35E−14	4.89E−14	5.36E−14	5.31E−14	5.55E−14	5.54E−14	5.37E−14	4.88E−14	5.34E−14
Eu-155	1.78E−15	1.48E−15	1.80E−15	1.73E−15	1.98E−15	1.94E−15	1.58E−15	1.46E−15	2.34E−15
Eu-156	5.53E−14	5.11E−14	5.58E−14	5.48E−14	5.78E−14	5.74E−14	5.46E−14	5.09E−14	5.42E−14
Hf-181	2.08E−14	1.88E−14	2.08E−14	2.03E−14	2.20E−14	2.18E−14	2.05E−14	1.87E−14	2.25E−14
Ta-182	5.54E−14	5.01E−14	5.51E−14	5.48E−14	5.72E−14	5.71E−14	5.58E−14	5.04E−14	5.53E−14
W-187	1.80E−14	1.65E−14	1.82E−14	1.78E−14	1.91E−14	1.89E−14	1.76E−14	1.63E−14	1.88E−14
Pb-210	3.56E−17	2.32E−17	3.59E−17	3.31E−17	4.20E−17	4.02E−17	2.84E−17	2.39E−17	4.66E−17
Pb-212	5.23E−15	4.48E−15	5.27E−15	5.14E−15	5.60E−15	5.51E−15	5.00E−15	4.58E−15	6.11E−15
Bi-212	4.47E−15	4.08E−15	4.51E−15	4.45E−15	4.70E−15	4.65E−15	4.39E−15	4.09E−15	4.48E−15
Ra-224	3.92E−16	3.39E−16	3.95E−16	3.86E−16	4.19E−16	4.11E−16	3.78E−16	3.48E−16	4.47E−16
Ra-226	2.67E−16	2.34E−16	2.66E−16	2.59E−16	2.84E−16	2.80E−16	2.49E−16	2.33E−16	3.18E−16
Ac-228	3.69E−14	3.39E−14	3.71E−14	3.67E−14	3.83E−14	3.83E−14	3.75E−14	3.37E−14	3.70E−14
Th-228	6.83E−17	5.84E−17	6.89E−17	6.66E−17	7.39E−17	7.30E−17	6.30E−17	5.81E−17	8.60E−17
Th-231	3.53E−16	2.89E−16	3.55E−16	3.40E−16	3.89E−16	3.82E−16	3.11E−16	2.84E−16	4.59E−16
Th-232	5.66E−18	4.38E−18	5.60E−18	5.45E−18	6.27E−18	6.14E−18	4.96E−18	4.39E−18	7.46E−18
Th-234	2.60E−16	2.15E−16	2.63E−16	2.54E−16	2.90E−16	2.84E−16	2.31E−16	2.12E−16	3.47E−16
Pa-233	8.10E−15	6.99E−15	8.22E−15	8.07E−15	8.69E−15	8.56E−15	7.96E−15	7.20E−15	9.16E−15
U-232	7.37E−18	5.81E−18	7.25E−18	6.94E−18	8.07E−18	7.85E−18	6.44E−18	5.83E−18	9.16E−18
U-234	3.64E−18	2.73E−18	3.51E−18	3.31E−18	3.97E−18	3.83E−18	2.99E−18	2.73E−18	4.48E−18
U-235	5.91E−15	5.18E−15	5.91E−15	5.76E−15	6.28E−15	6.21E−15	5.52E−15	5.15E−15	7.07E−15
U-236	1.84E−18	1.26E−18	1.70E−18	1.57E−18	1.98E−18	1.88E−18	1.39E−18	1.27E−18	2.16E−18
U-237	4.43E−15	3.79E−15	4.46E−15	4.32E−15	4.83E−15	4.73E−15	4.05E−15	3.77E−15	5.49E−15
U-238	1.64E−18	1.18E−18	1.53E−18	1.43E−18	1.75E−18	1.68E−18	1.30E−18	1.19E−18	1.84E−18
Np-237	7.00E−16	5.83E−16	7.06E−16	6.76E−16	7.70E−16	7.58E−16	6.21E−16	5.74E−16	9.04E−16
Np-238	2.53E−14	2.37E−14	2.54E−14	2.53E−14	2.59E−14	2.62E−14	2.64E−14	2.32E−14	2.49E−14
Np-239	6.24E−15	5.40E−15	6.31E−15	6.15E−15	6.75E−15	6.63E−15	5.86E−15	5.43E−15	7.51E−15
Pu-236	1.67E−18	9.68E−19	1.38E−18	1.21E−18	1.72E−18	1.59E−18	1.07E−18	9.66E−19	1.65E−18
Pu-238	1.02E−18	4.76E−19	7.46E−19	6.04E−19	1.00E−18	8.97E−19	5.35E−19	4.72E−19	8.63E−19
Pu-239	2.46E−18	1.95E−18	2.36E−18	2.26E−18	2.63E−18	2.55E−18	2.14E−18	1.95E−18	2.78E−18
Pu-240	1.06E−18	5.27E−19	8.10E−19	6.72E−19	1.07E−18	9.61E−19	5.92E−19	5.25E−19	9.47E−19
Pu-242	4.25E−18	3.61E−18	4.05E−18	3.94E−18	4.40E−18	4.28E−18	3.81E−18	3.56E−18	4.09E−18
Am-241	5.32E−16	3.92E−16	5.34E−16	5.11E−16	6.15E−16	5.92E−16	4.64E−16	3.99E−16	7.14E−16
Am-242	4.41E−16	3.82E−16	4.45E−16	4.31E−16	4.85E−16	4.76E−16	3.92E−16	3.72E−16	5.71E−16
Am-242m	1.06E−17	7.24E−18	9.52E−18	8.62E−18	1.13E−17	1.07E−17	7.81E−18	7.12E−18	1.15E−17
Am-243	1.55E−15	1.24E−15	1.56E−15	1.52E−15	1.70E−15	1.69E−15	1.38E−15	1.24E−15	2.10E−15
Cm-242	1.20E−18	4.72E−19	8.16E−19	6.05E−19	1.18E−18	1.02E−18	5.33E−19	4.60E−19	8.48E−19
Cm-243	4.55E−15	3.93E−15	4.60E−15	4.49E−15	4.91E−15	4.83E−15	4.29E−15	3.97E−15	5.43E−15
Cm-244	1.65E−18	9.99E−19	1.32E−18	1.14E−18	1.65E−18	1.51E−18	1.08E−18	9.81E−19	1.32E−18
Cm-245	3.35E−15	2.92E−15	3.38E−15	3.28E−15	3.65E−15	3.59E−15	3.02E−15	2.85E−15	4.27E−15
Cm-247	1.25E−14	1.12E−14	1.25E−14	1.23E−14	1.32E−14	1.31E−14	1.24E−14	1.12E−14	1.34E−14

Full list for all radionuclides can be found at the electronic supplement

Similarly to Fig. 4 for monoenergetic coefficients, the nuclide-specific uterus doses of “Katja” were compared to the nuclide-specific total body doses of the fetus. The comparisons revealed that these agree within 6% for most of the radionuclides. For ^131^I, ^132^I, ^133^I, ^134^Cs, ^136^Cs, ^137m^Ba, ^140^Ba, ^132^Te, ^140^La, ^95^Zr, ^95^Nb, ^95^Mo, ^103^Ru for example, and soil contamination, the ratio of dose to the uterus to the fetal total body dose was 1.04–1.06. For ^137^Cs and ^133^Xe, the ratio was found to be 1.09. Larger discrepancies were observed for nuclides emitting low-energy photons and beta emitters generating low-energy bremsstrahlung photons, because the latter are attenuated before reaching the fetus. Note, that the comparisons refer to the radionuclide without progeny.

Moreover, Table 6 reveals that the air kerma coefficients and the detriment-weighted dose coefficients of “Katja” are rather similar to the uterus (“Katja”) and fetus total body coefficients for many radionuclides—demonstrating that environmental coefficients are relatively independent of the exact organ position inside a body having a specific size. Furthermore, as an initial response to an accident, rough estimates of fetal doses can be done by the measured data of air kerma.

It should be noted that previous studies of Petoussi et al. (2012) did not include the contribution of prompt and delayed photons and beta emissions following spontaneous fission: there are 28 radionuclides (ICRP 2008), i.e., ^254^Cf, ^250^Cm, ^252^Cm, ^256^Fm, ^244^Pu etc. which undergo spontaneous fission resulting in emission of fast neutrons. Although the dose contribution from the fast neutrons is regarded to be negligible and is not considered, the photon contribution of spontaneous fission to the organ doses is included in the present study.

## Conclusions

This work provides a dataset of organ dose rate coefficients of a pregnant female and its fetus at the 24^th^ week of pregnancy to be used for the assessment of external dose from environmental exposure for two typical environmental conditions. The computations of the dose coefficients were based on methodologies previously developed. The equivalent dose coefficients are given as Sv s^−1^ per source activity concentration. Air kerma rate coefficients at 1 m above ground are also given and can be used to normalize the organ dose rate coefficients to rates of this measurable quantity.

In real environments, the conditions may differ from the postulated conditions, and therefore, the dose coefficients would vary. The potential factors which may affect the dose coefficients include, in case of accidental release, location, distance from the site, duration of the release, deposition pathways, chemical form of released radionuclides, weather conditions at the time of the release such as wind direction and any rainfall or snowfall occurring during the passage of the plume. The levels of deposition may also vary greatly from one area to another. For a routine or extended release, wind direction can be expected to vary over time. In the longer term, rainfall, snowfall, and weathering will allow penetration of deposited radionuclides into soil and some migration via water pathways or through resuspension (WHO 2012; ICRP 2020). Generally, in the longer term, one or a few radionuclides will dominate as the principal contributors to human exposure (ICRP 2009b). In addition, the habits of the population for whom doses are being assessed would affect the doses received, as well as individual characteristics such as body size, stage of pregnancy and posture.

Moreover, in real situation, people are not always standing on contaminated ground, as they spend much time indoors and are lying during nights. External doses can be significantly lower indoors than outdoors due to the shielding effects of the building. The assessment of the real dose assumes a location factor (referred also as shielding factor, dose reduction factor or protection factor) in the range of 0.005–0.4 that reflects the amount of shielding from external radiation provided by housing according to the geographical distribution and building material and an assumed occupancy factor of 66%, i.e., two thirds of the time per day spent indoors (IAEA 2000). This can obviously vary a lot according to country, weather conditions, profession, and habits of the individuals.

This article provides the basic data for the assessment of fetal doses for exposure situations, involving naturally occurring radionuclides in the environment, anthropogenic radionuclides from routine or accidental releases, for prospective and retrospective purposes. The methodology for the realistic assessment takes into account geographical and population specific characteristics such as climate, terrain, agricultural production, infrastructure, i.e., housing and culture.

## Electronic supplementary material

Below is the link to the electronic supplementary material.

Supplementary file 1 (XLSX 183 KB)

Supplementary file 2 (XLSX 361 KB)

Supplementary file 3 (XLSX 40 KB)

Supplementary file 4 (XLSX 140 KB)

Supplementary file 5 (XLSX 351 KB)

Supplementary file 6 (XLSX 44 KB)
